# A plant virus satellite RNA directly accelerates wing formation in its insect vector for spread

**DOI:** 10.1038/s41467-021-27330-4

**Published:** 2021-12-06

**Authors:** Wikum H. Jayasinghe, Hangil Kim, Yusuke Nakada, Chikara Masuta

**Affiliations:** 1 Graduate School of Agriculture, Hokkaido University, Kita-ku, Kita 9 Nishi 9, Sapporo, 060-8589 Japan; 2 Department of Agricultural Biology, Faculty of Agriculture, University of Peradeniya, Peradeniya, Sri Lanka

**Keywords:** Plant molecular biology, Biotic, Coevolution

## Abstract

Cucumber mosaic virus (CMV) often accompanies a short RNA molecule called a satellite RNA (satRNA). When infected with CMV in the presence of Y-satellite RNA (Y-sat), tobacco leaves develop a green mosaic, then turn yellow. Y-sat has been identified in the fields in Japan. Here, we show that the yellow leaf colour preferentially attracts aphids, and that the aphids fed on yellow plants, which harbour Y-sat-derived small RNAs (sRNAs), turn red and subsequently develop wings. In addition, we found that leaf yellowing did not necessarily reduce photosynthesis, and that viral transmission was not greatly affected despite the low viral titer in the Y-sat-infected plants. Y-sat-infected plants can therefore support a sufficient number of aphids to allow for efficient virus transmission. Our results demonstrate that Y-sat directly alters aphid physiology via Y-sat sRNAs to promote wing formation, an unprecedented survival strategy that enables outward spread via the winged insect vector.

## Abstract (web summary)

The cucumber mosaic virus is accompanied by short RNA molecules, satellite RNAs. This study shows that leaves infected with Y-satellite RNA preferentially attract aphids and manipulate aphid physiology to promote their spread to neighbouring plants.

## Introduction

**Fig. 1:**
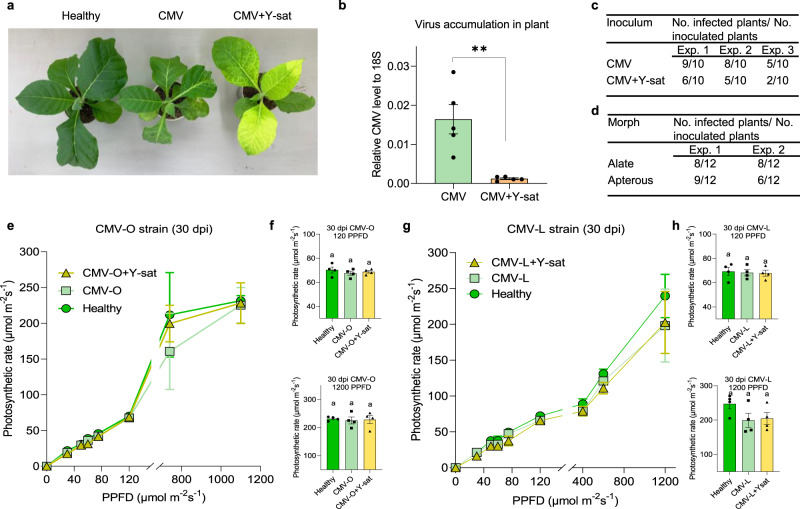
Effect of Y-satellite (Y-sat) infection on tobacco growth and virus transmission by aphids. **a** Yellowing was induced in (cucumber mosaic virus [CMV] + Y-sat)-infected plants but not the CMV-infected or healthy at 14 d.p.i. Note that [CMV + Y-sat]-infected plants had few abnormally shaped leaves. **b** Each data point represents the relative CMV levels in CMV- and [CMV + Y-sat]-infected plants determined by qPCR. Data are mean values (±SE) (two-sided *t* test, *n* = 5, *P* = 0.0037). **P < 0.01. **c** Efficiency of aphid transmission of virus from CMV-infected and [CMV + Y-sat]-infected plants to healthy plants (*χ*^2^ test, *P* = 0.0352). **d** CMV transmission efficiency by apterous and alate aphids (*χ*^2^ test, *P* > 0.9999). **e**–**h** Mean (±SE) photosynthetic rate in CMV-O (or -L)-infected, [CMV + Y-sat]-infected and healthy leaves at 30 d.p.i. at various photosynthetic proton flux densities (PPFD) µmol m^−2^ s^−1^. Individual data points are mean values of each plant (**e**, *n* = 4; **g**, *n* = 3). **f**, **h** Mean (±SE) photosynthetic rate among infection types were analysed at 120 and 1200 PPFD by one-way ANOVA with Tukey’s multiple comparison test (*n* = 4, values followed by the same letters are not significantly different at *P* < 0.05). 120 PPFD: CMV-O-infected (*P* = 0.5914); CMV-L-infected (*P* = 0.9293). 1200 PPFD: CMV-O-infected (*P* = 0.9256); CMV-L-infected (*P* = 0.1695). Source data are provided as a Source Data file.

Satellite RNAs (satRNAs), which are associated with helper viruses, belong to the group of subviral agents. Cucumber mosaic virus (CMV) satRNAs ranging from 300 to 400 nucleotides in size are regarded as long noncoding RNAs^1^. CMV satRNAs typically reduce the viral titre and are transmitted with CMV by aphids. They rely on the helper virus CMV for their replication and encapsidation^2,3^. CMV satRNAs generally have a negative effect on the accumulation and vector transmission of the virus and often alter viral symptoms^3,4^. Y-satellite RNA (Y-sat) has been discovered in the fields in Japan in 1981^5^ and still sporadically found in the fields. It induces bright yellow symptoms on *Nicotiana* species, although it can somehow attenuate CMV-induced symptoms^6^. In our previous investigation of persistence between four CMV satRNAs in mixed inoculation, we found that Y-sat is less competitive with other CMV satRNAs^7^. For the molecular mechanism for the yellow symptom induction by Y-sat, we previously demonstrated that Y-sat turned green mosaics induced by CMV to bright yellow (Fig. 1a) through the downregulation of the expression of the *ChlI* gene essential for chlorophyll synthesis by specific small RNAs (sRNAs) derived from Y-sat^6^.

CMV is nonpersistently transmitted; it is considered to bind loosely to a receptor in the tip of the aphid stylet and retain for a short period^8–10^. CMV satRNAs are dependent on a helper virus for their transmission, being encapsidated into viral particles. Base on the fact that Y-sat has not disappeared in the fields until today, it seems to be efficiently transmitted by aphids. This transmission efficiency of [CMV + Y-sat] by the vector aphids led us to believe that Y-sat might actively attract aphids by causing the leaf yellowing. The epidemiology of a virus with a wide host range is closely related to the behaviour of the aphid vector^11^. Furthermore, the conspicuous presence of red aphids on Y-sat-infected tobacco led us to suspect that Y-sat may influence aphid physiology and thereby its behaviour. In this paper, we present a survival strategy in which Y-sat can apparently control the helper virus, host plant and insect vector.

## Results and discussion

### Onward transmission of Y-sat and CMV

Despite the bright yellowing, leaves of Y-sat-infected tobacco plants had attenuated symptoms merely due to the greatly reduced CMV accumulation; the relative CMV level in the Y-sat-infected plants was 13.5 times lower than in plants infected only with CMV (*t* test, *P* = 0.0037) (Fig. 1b). Typically, the efficiency of plant virus transmission is positively correlated with the levels of the virus^12^. Because the presence of the satRNAs reduces the accumulation of the helper virus, it negatively affects the transmission of the helper virus^13^. In light of these findings, we predicted that the efficiency of aphid transmission of CMV + Y-sat would be greatly reduced and that Y-sat might disappear with time^13^. Surprisingly, in the aphid transmission experiments using ten aphids per test plant showed a relatively high transmission efficiency (43.3% for [CMV strain O (CMV-O) + Y-sat]-infected plants vs 73.3% for CMV-infected plants, *χ*^2^ = 4.3886, d.f. = 1, *P* = 0.0362) (Fig. 1c and Supplementary Fig. 1a). The virus transmission efficiency was not significantly different between alate and apterous aphid (*χ*^2^ = 0, d.f. = 1, *P* = 1) (Fig. 1d). Although the efficiency was reduced by 30% compared to the control, according to Markov chain-based epidemiological dynamics^11^, 43.3% transmission efficiency is ample for CMV spread and persistence in nature.

To determine the reason for high level of aphid transmission efficiency, we measured the CMV levels in individual aphids feeding on CMV-infected and [CMV + Y-sat]-infected tobacco plants using reverse transcription-quantitative real-time PCR. The results showed that the average CMV level in a single aphid after feeding on [CMV + Y-sat]-infected plants was only 2.6 times lower than in those that fed on CMV-infected plants (*t* test, *P* = 0.0006) (Supplementary Fig. 1b) We thus hypothesized that the aphid stylet was the limiting factor and had a threshold for acquiring CMV. Therefore, the CMV levels in a single aphid can explain the relatively efficient aphid transmission of [CMV + Y-sat] even though the levels of CMV were extremely low in the infected plants.

### Y-sat represses adverse effects on host

The yellowed leaves of Y-sat-infected plants would be expected to have less chlorophyll and thus reduced photosynthesis; thus, Y-sat infection might eventually kill the plants. However, in our preliminary observations, Y-sat-infected yellow plants actually grew better than the CMV-infected plants, and growth was even comparable to the healthy plants under some stress conditions such as drought (analysis of variance (ANOVA), Tukey, *P* = 0.0115) (Supplementary Fig. 2a, b). We thus tested the effect of CMV and [CMV + Y-sat] infection on photosynthesis in tobacco by measuring the photosynthetic rate under different light intensities at 15 days post inoculation (d.p.i.) and 30 d.p.i. A severe (CMV-O) and a mild (CMV strain L [CMV-L]) strain were used to determine whether photosynthesis varied depending on the CMV strain. Although photosynthesis in the Y-sat-infected plants was reduced at an early stage of infection (15 d.p.i.) (ANOVA, Tukey, CMV-O-infected, 120 photosynthetic photon flux density (PPFD), *P* = 0.003, 1200 PPFD, *P* = 0.3769; CMV-L-infected, 120 PPFD, *P* = 0.8239, 1100 PPFD, *P* = 0.0259) (Supplementary Fig. 2c–h), by 30 d.p.i. when viral symptoms had become attenuated by plant’s defence mechanisms, we found no significant difference among the healthy, CMV- and [CMV + Y-sat]-infected plants (ANOVA, Tukey, CMV-O-infected, 120 PPFD, *P* = 0.5914, 1200 PPFD, *P* = 0.9256; CMV-L-infected, 120 PPFD, *P* = 0.9293, 1200 PPFD, *P* = 0.1695) (Fig. 1e–h). Nor did they differ significantly in stomatal conductance (Supplementary Fig. 3a, b). These results suggest that the yellow plants can maintain a relatively high rate of photosynthesis unless they are severely damaged. In addition, chloroplasts in the Y-sat-infected tissues are ultrastructurally indistinguishable from those in the healthy plants, suggesting that the chloroplasts are not seriously damaged by Y-sat infection^14^.

After infection with a severe CMV strain, tobacco plants often develop mosaics, stunting and leaf malformation, which affect plant growth and survival. A heavy viral load often leads to damaged chloroplast structures in the leaf^15^. In contrast, Y-sat infection did not cause much damage to the chloroplasts; instead, virus symptoms were attenuated, although the leaves became yellow^6^. Therefore, we presume that the leaf yellowing induced by Y-sat does not seriously affect photosynthesis.

### Red morphs in the aphid life cycle

**Fig. 2:**
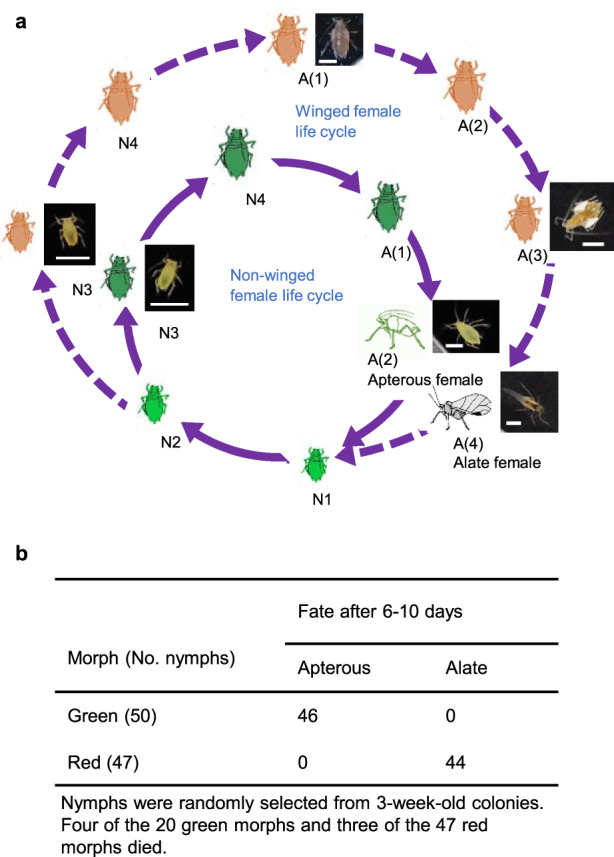
Wing formation by *Myzus persicae* is linked to body colour. Red and green morphs of aphids from the same colony descended from a single mother have different fates in their life cycle. **a** Viviparous life cycle of green and red morphs. A: adult, N: nymph. The red and green nymphs were from uninfected stock plants. The aphid depiction was generated from our original photos. **b** Differences in fates between green and red morphs. All green morphs developed into apterous adults; all red morphs developed into alate adults. The adult stage could be divided into A(1)–A(4) based on changes in body colour, body size and wing-bud size. For detailed information, see the Supplementary Note 1 and ‘Methods’. Scale bars = 1 mm.

We first noticed that the aphids on Y-sat-infected plants produced significantly more red nymphs than those on CMV-infected plants; this initial observation promoted us to conduct this study. Our observations of the life cycle of aphids from a single mother revealed that these red nymphs finally developed into viviparous alates (Fig. 2a). We observed an association between colour and wing formation in *Myzus persicae*. The green nymphs finally developed into apterous adults (Fig. 2b, Supplementary Note 1 and ‘Methods’). Regarding aphid colour changes, the red pigmentation gene in *Acyrthosiphon pisum* was laterally transferred from a fungus^16^. *Rickettsiella* infection also affected red coloration in *A. pisum*^17^. The red morph is believed to provide an advantage for avoiding parasitoids^18^, but little is known about a link between the red morph and wing formation.

### Push–pull strategy of aphid control by Y-sat

**Fig. 3:**
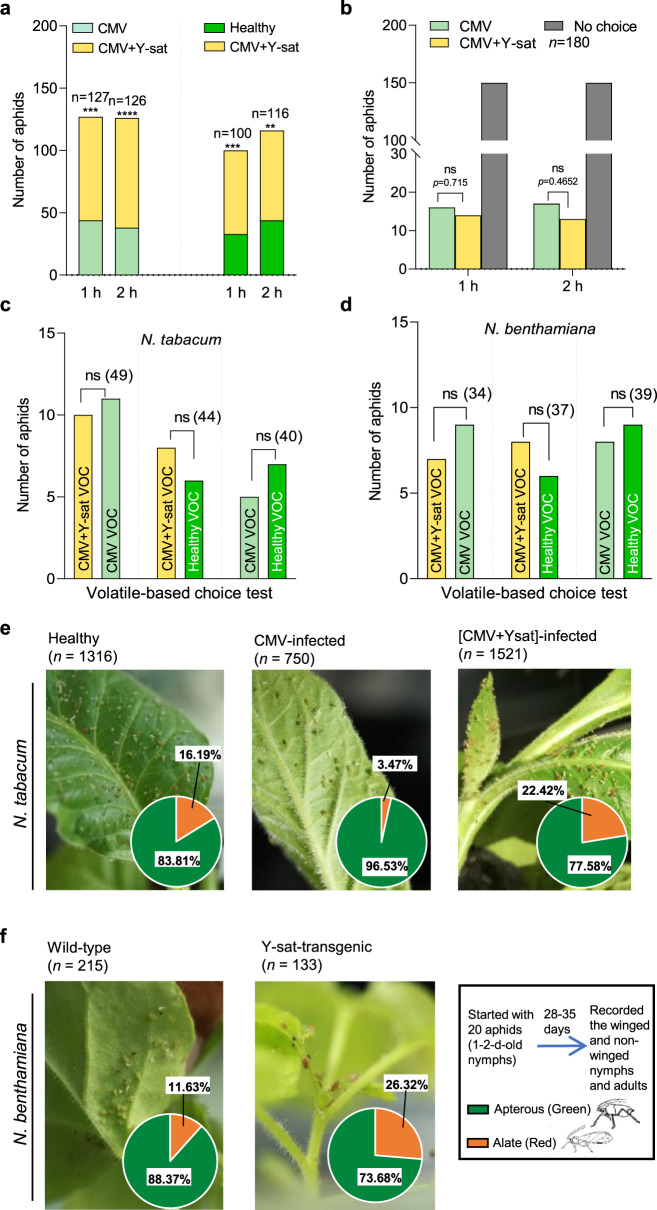
Aphids are attracted to Y-sat-infected plants and produce more alate adults. All the statistical analysis in Fig. 3 was performed using *χ*^2^ test. **a** Mean percentages (±SE) of aphids attracted to CMV-infected, [CMV + Y-sat]-infected or healthy *N. tabacum* at 1 and 2 h after aphid transfer. CMV-infected vs [CMV + Y-sat]-infected (1 h, *n* = 127, *P* = 0.0005; 2 h, *n* = 126, *P* < 0.0001), and healthy vs [CMV + Y-sat]-infected (1 h, *P* = 0.0007; 2 h*, P* = 0.0093). **b** Mean percentages (±SE) of aphids attracted to CMV-infected, [CMV + Y-sat]-infected or healthy *N. tabacum* at 1 and 2 h after aphid transfer under dark conditions. For statistics, *χ*^2^ test was used. CMV-infected vs [CMV + Y-sat]-infected (1 h, *n* = 180, choice vs no choice, *P* < 0.0001; CMV vs [CMV + Y-sat], *P* = 0.715) (2 h, *n* = 180, choice vs no choice, *P* < 0.0001; CMV vs [CMV + Y-sat], *P* = 0.4652). **c**, **d** Number of aphids attracted to volatile compounds from [CMV + Y-sat]-infected, CMV-infected or healthy *Nicotiana* species in Y-tube choice experiment. **c** *N. tabacum* [CMV + Y-sat]-infected vs CMV-infected (*n* = 49, *P* = 0.8273); [CMV + Y-sat]-infected vs healthy (*n* = 44, *P* = 0.593); healthy vs CMV-infected (*n* = 40, *P* = 0.5637). **d** *N. benthamiana* [CMV + Y-sat]-infected vs CMV-infected (*n* = 34, *P* = 0.6171); [CMV + Y-sat]-infected vs healthy (*n* = 37, *P* = 0.593); healthy vs CMV-infected (*n* = 39, *P* = 0.8084). For the statistical significance (**a**–**d**), the symbols **, ***, **** are significance levels of *P* < 0.01, *P* < 0.001 and *P* < 0.0001, respectively. ns, not significant. **e** Mean percentages of alate and apterous morphs on healthy (*n* = 1316), CMV-infected (*n* = 750) and [CMV + Y-sat]-infected (*n* = 1521) *N. tabacum*. Statistical analysis for three samples was performed using *χ*^2^ test (three samples, *P* < 0.0001; [CMV + Y-sat] vs CMV, *P* < 0.0001; [CMV + Y-sat] vs healthy, *P* < 0.0001; CMV vs healthy, *P* < 0.0001). **f** Mean percentages of alate and apterous morphs of aphid populations infesting wild-type (*n* = 215) or transgenic (*n* = 133) *N. benthamiana* plants expressing Y-sat dsRNA (dsY-sat). Statistical analysis for two samples was performed using *χ*^2^ test (two samples, *P* = 0.0007). The results of the *χ*^2^ tests in Fig. 3 were also confirmed by another statistical method, a hypothesis test for one-sample (or two-sample) proportion in R (see the data set in Supplementary Methods 1). Source data are provided as a Source Data file.

Vector attraction to an infected host plant and subsequently to another host plant is vital for the spread of Y-sat. Previously it has been shown that *M. persicae* is generally attracted to yellow colour^19,20^. Therefore, we first tested whether *M. persicae* is attracted to Y-sat-infected yellow and healthy tobacco plants using a pairwise bioassay (Supplementary Fig. 4a and see ‘Methods’) between CMV- and [CMV + Y-sat]-infected plants. The proportion of aphids attracted to [CMV + Y-sat]-infected plants was significantly higher after a 1- and 2-h exposure (1 h, *χ*^2^ = 11.976, d.f. = 1, *P* = 0.0005; 2 h, *χ*^2^ = 19.841, d.f. = 1, *P* < 0.0001) (Fig. 3a). In assays pairing healthy and [CMV + Y-sat]-infected plants, significantly more aphids also selected the Y-sat-infected plants (1 h, *χ*^2^ = 11.56, d.f. = 1, *P* = 0.0007*;* 2 h, *χ*^2^ = 6.7586, d.f. = 1, *P* = 0.0093) (Fig. 3a). To further confirm the yellow attraction of aphid, we performed an aphid attraction bioassay in the dark. As we expected, the majority (~83.3%) of aphid did not move to both CMV- and [CMV + Y-sat]-infected plants (*χ*^2^ = 80, d.f. = 1, *P* < 0.0001). As a result, there was no significant difference between the two plants (1 h, *χ*^2^ = 0.1333, d.f. = 1, *P* = 0.715; 2 h, *χ*^2^ = 0.5333, d.f. = 1, *P* = 0.4652) (Fig. 3b), suggesting that aphids rely on their eyesight for probing plants. In a given ecosystem, aphids will be quickly attracted to yellow plants, spending less time and saving energy to find a suitable host; this yellow attraction can be regarded as a pull strategy of Y-sat.

To investigate whether volatile organic compounds (VOCs) contribute to attracting aphids to Y-sat-infected plants, we designed an olfactory bioassay (Supplementary Fig. 4b and see ‘Methods’) using different paired combinations of healthy, CMV- and [CMV + Y-sat]-infected tobacco. For this assay, we used two *Nicotiana* species, *N. tabacum* (Fig. 3c) and *N. benthamiana* (Fig. 3d). In each set of experiments, only ~40% of the aphids were attracted to either of the tested plants; the remaining aphids had no preference in both *N. tabacum* ([CMV + Y-sat]-infected vs CMV-infected, *χ*^2^ = 0.0476, d.f = 1, *P* = 0.8273; [CMV + Y-sat]-infected vs healthy, *χ*^2^ = 0.2857, d.f. = 1, *P* = 0.593; healthy vs CMV-infected, *χ*^2^ = 0.3333, df = 1, *P* = 0.5637) and *N. benthamiana* ([CMV + Y-sat]-infected vs CMV-infected, *χ*^2^ = 0.25, d.f. = 1, *P* = 0.6171); [CMV + Y-sat]-infected vs healthy, *χ*^2^ = 0.2857, d.f. = 1, *P* = 0.593; healthy vs CMV-infected, *χ*^2^ = 0.0588, d.f. = 1, *P* = 0.8084). We thus confirmed that neither CMV infection nor Y-sat infection induces a scent-dependent attraction to aphids. According to a previous report, CMV infection can alter the VOCs in tobacco but does not alter aphid attraction to the plant^21^, supporting our hypothesis that both species infected with [CMV + Y-sat] can attract a significantly higher number of aphids merely due to the yellow colour induced by Y-sat.

**Fig. 4:**
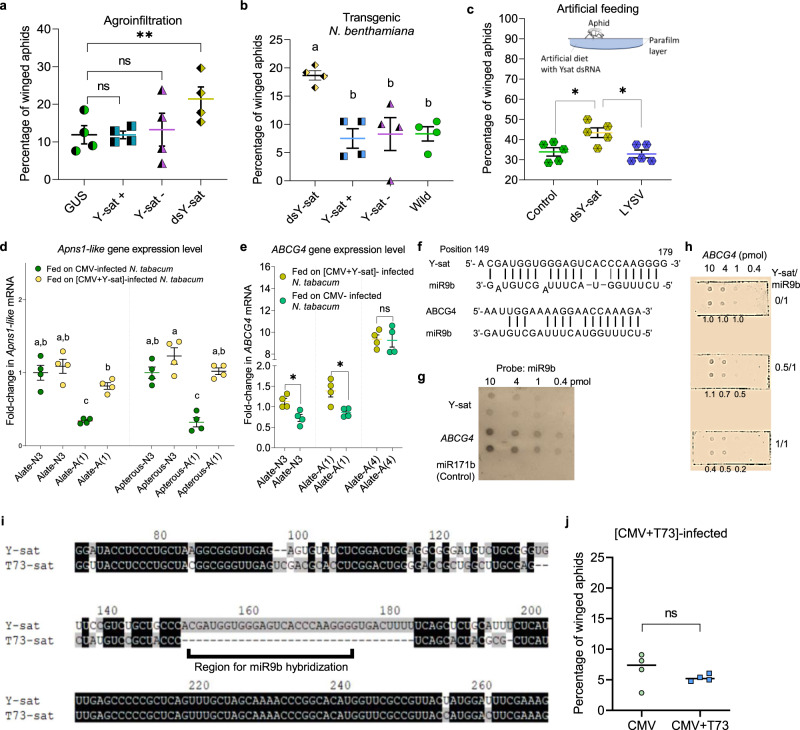
Effect of Y-sat on alate formation. **a** Mean percentages of winged aphids in *N. benthamiana* transiently expressing Y-sat dsRNA (dsY-sat), plus-sense (Y-sat+) and minus-sense RNA (Y-sat−) relative to respective controls (one-way ANOVA, Dunnett’s test, *n* = 4, *P* = 0.0026). *Agrobacterium* carrying pBE2113-GUS was used as a control. **b** Mean percentages of winged aphids on transgenic plants expressing either dsY-sat, Y-sat+ or Y-sat− (one-way ANOVA, Tukey’s test, *n* = 4, *P* = 0.0030). **c** Proportion of alate morphs fed on diet containing dsY-sat (one-way ANOVA, Tukey’s test, *n* = 5, *P* = 0.0085). Controls: leek yellow stripe virus dsRNA; H_2_O. **d** Relative expression of *Apns1-like* in aphids fed on CMV-infected or [CMV + Y-sat]-infected plants. Third instars (N3) and early adult [A(1)] are as explained in Fig. 2a (one-way ANOVA, Tukey’s test, *n* = 4, *P* < 0.0001). **e** Relative expression of *ABCG4* in N3 (two-sided *t* test, *n* = 4, *P* = 0.0122), A(1) (two-sided *t* test, *n* = 4, *P* = 0.0150) and late adults [A(4)] (two-sided *t* test, *n* = 4, *P* = 0.8923) fed on CMV-infected or [CMV + Y-sat]-infected tobacco plants. Each data point in Fig. 4e represents the level of mRNA isolated from aphids [N3, ten individuals; A(1) and A(4), one individual]. **f** Complementarity between miR9b and Y-sat or *ABCG4*. **g** RNA dot-blot hybridization. RNA oligonucleotides of Y-sat and *ABCG4* complementary to miR9b shown in Fig. 4g were synthesized, blotted onto a nylon membrane (0.4–10 pmol) and probed by Dig-labelled miR9b. **h** Competitive hybridization assay. When *ABCG4* RNA was probed with Dig-miR9b, Y-sat RNA was added at molar ratios indicated on right. Results were the same in two repeated experiments. Values on membrane are relative values determined using ImageJ, without Y-sat was set to 1.0. **i** RNA sequence alignment between Y-sat and T73 satellite (T73-sat). The complementary region to miR9b in Y-sat is indicated. **j** Mean percentages of alate morphs infesting CMV-infected or [CMV + T73]-infected tobacco. (GLM test in R, *n* = 4, *z* = −0.473, *P* = 0.636). Each data point in (**a**–**c**) and (**j**) represents the percentage of winged aphids. Values followed by the same letters in (**b**) and (**d**) are not significantly different. **, *P* < 0.01; *, *P* < 0.05; ns, not significant. Source data are provided as a Source Data file.

When we observed and counted the aphids, [CMV + Y-sat]-infected and Y-sat-transgenic plants had significantly more red nymphs than on either healthy or CMV-infected plants (healthy vs CMV-infected vs [CMV + Y-sat]-infected, *χ*^2^ = 133.11, d.f. = 2, *P* < 0.0001; wild type vs Y-sat-transgenic, *χ*^2^ = 11.415, d.f. = 1, *P* = 0.0007) (Fig. 3e, f). So we wondered whether Y-sat directly regulated aphid development. To test this idea, we first expressed the Y-sat sequence as plus-sense, negative-sense, or double-stranded (ds)RNA in a transient expression assay using agroinfiltration of *N. benthamiana* leaves, and then reared the aphids on the agroinfiltrated leaves (Fig. 4a and see ‘Methods’). We found that alate aphids were most numerous on leaves expressing Y-sat dsRNA (ANOVA, Dunnett, *P* = 0.0031). We thus hypothesized that Y-sat dsRNA might induce alate development. We then generated transgenic *N. benthamiana* plants using the three Y-sat expression constructs used for the transient expression assay. When aphids were raised on these three types of transgenic plants, aphids turned red most frequently on the transgenic plants expressing Y-sat dsRNA. These results also indicate that neither the Y-sat plus-sense nor the negative-sense RNAs affect aphid development (ANOVA, Tukey, *P* = 0.0030) (Figs. 3f and 4b). Finally, to rule out the involvement of *Agrobacterium* or any host factors, we fed aphids with a synthetic diet containing Y-sat dsRNA and, as expected, Y-sat dsRNA induced more alate aphids (ANOVA, Tukey, *P* = 0.0085) (Fig. 4c), indicating that Y-sat dsRNA explicitly affects alate production of *M. persicae* (Supplementary Fig. 5). Y-sat thus seems to be actively inducing the formation of alate aphids and thus their spread, which could be regarded as a push strategy deployed by Y-sat.

### Y-sat dsRNA modulates wing formation

To elucidate the mechanism of this phenomenon, we first analysed how Y-sat dsRNA is processed in the aphid by sRNA-seq and found that long small interfering RNAs (siRNAs) (25–33 nt) were mainly produced despite that the major aphid sRNAs were 22–25 nt (Supplementary Fig. 6a–f). Because aphid ingestion of dsRNA from plants can induce gene silencing in aphids^22^, we hypothesized that these long siRNAs might be virus-derived P-element-induced wimpy testis (*P*IWI)-interacting RNAs (piRNAs) (vd-piRNAs), which were discovered by Wu et al.^23^ in *Drosophila* cells infected with RNA viruses. Regarding the function of vd-piRNAs, Schnettler et al.^24^ reported antiviral properties of vd-piRNAs in mosquito cells infected with semliki forest virus. According to Morazzani et al.^25^, vd-piRNAs are produced through a non-canonical pathway, contrary to conventional piRNAs against transposons.

So, how can Y-sat-derived piRNAs (Ysd-piRNAs) induce wing formation? Two genes were recently discovered to be essential for wing formation. One is *Apns1* of densovirus origin^26^; expressed early in the life cycle, it triggers wing formation and then gradually decreases in expression. The other is the *ABCG4* gene^27^, which is expressed late in the life cycle to control the physiology of wing formation. By a sequence homology search against the Apns1 protein with BLASTP in NCBI, we identified the *Apns1* homologue in *M. persicae* (*Apns1-like*), which was closely related to both *Apns1* and *Dysaphis plantaginea* densovirus in our phylogenetic tree (Supplementary Fig. 7). The expression of the *Apns1-like* gene was increased to a relatively high level already at the nymph stage (alate-N3 and apterous-N3) fed on both CMV- and [CMV + Y-sat]-infected tobacco plants (ANOVA, Tukey, *P* < 0.0001) (Fig. 4d). Then, the gene expression decreased in the individuals fed on CMV-infected plants but remained at a similar level on [CMV + Y-sat]-infected plants as the aphid instar stage progressed. In addition, the gene expression levels became relatively higher in the individuals [alate-A(1) and apterous-A(1)] fed on [CMV + Y-sat]-infected plants than those on CMV-infected plants (ANOVA, Tukey, *P* < 0.0001) (Fig. 4d). The same results were obtained when transgenic tobacco plants expressing Y-sat dsRNA were used instead of Y-sat-infected plants (ANOVA, Tukey, *P* < 0.0001) (Supplementary Fig. 8a). Similarly, the expression levels of the *ABCG4* gene were significantly higher in the alate aphids [alate-N3 and alate-A(1)] fed on [CMV + Y-sat]-infected plants than those on CMV-infected plants (*t* test, alate-N3, *P* = 0.0122; alate-A(1), *P* = 0.0150) (Fig. 4e). Just like the *Apns1-like* gene, we obtained similar results when we used transgenic plants expressing Y-sat dsRNA (*t* test, *P* = 0.0014, 0.0737, 0.0029 and 0.0519) (Supplementary Fig. 8b). Based on these results, we believe that Y-sat can indeed upregulate the expression of two genes essential for wing formation at appropriate times in the aphid life cycle.

### Y-sat uses silencing to induce wings

We then focused on the fact that the expression of the *ABCG4* gene is regulated by miR9b^27^. As shown in Fig. 4f, we found that the Y-sat central sequence showed some complementarity to miR9b. In our sRNA-seq, we could identify both the reads of the mature miR9b and the reads of Ysd-piRNA derived from the Y-sat central sequence (Supplementary Fig. 6g, h); therefore, Ysd-piRNAs may interfere with miR9b in binding to *ABCG4* messenger RNA (mRNA). To explore this possibility, we synthesized RNAs of miR9b, *ABCG4* mRNA and Y-sat RNA for RNA dot-blot hybridizations. Although we found that miR9b bound to Y-sat more weakly than to *ABCG4* mRNA in vitro (Fig. 4g), our competitive hybridization assay suggested that Y-sat somehow interfered with the binding of miR9b to *ABCG4* mRNA even at an equivocal molar ratio (Fig. 4h). Therefore, we believe that Ysd-piRNA may upregulate the expression of the *ABCG4* gene by competing with *ABCG4* mRNA for binding to miR9b. We here propose a possible molecular mechanism by which Y-sat dsRNA can induce aphid wing formation by upregulating the expression of the *ABCG4* gene. To go one step further, we tested alate induction using a different satellite RNA (T73-sat), which does not contain any complementary sequence to miR9b (Fig. 4i). The result showed that T73-sat could not induce alate (generalized linear model (GLM) test in R, *z* = −0.473, *P* = 0.636) (Fig. 4j) as Y-sat could, suggesting that alate induction is promoted by a specific mechanism mediated by the unique sequence in Y-sat. In this study, we hypothesized that the expression of the *Apns1-like* and *ABCG4* genes promotes the induction of wing formation in aphids. However, many questions still remain to be answered toward fully understanding the mechanism(s); e.g., the *ABCG4* mRNA and Y-sat siRNAs must be present in the same aphid cells according to our hypothesis.

We also tested the plant hormone jasmonic acid (JA), sugar levels in the phloem sap and aphid population size as factors that might alter the alate population. To analyse the JA and sugar levels, rather old leaf tissues were used because viral symptoms are somehow attenuated by host silencing and the viral and Y-sat levels are relatively at steady states. JA acts as a central signal in plant defence against herbivore insects^28,29^. The composition of phloem sap also plays a major role in the likelihood that an aphid will continue feeding on a plant; an increase in sugar content in the phloem sap can alter the aphid behaviour on plants^30^. We thus measured the levels of sucrose and d-glucose in healthy and infected plant leaves. When we determined the levels of JA, sucrose and glucose in healthy, CMV- and [CMV + Y-sat]-infected plants, there was no significant difference among the three plants (ANOVA, JA, *P* = 0.9707; CMV-O-glucose, *P* = 0.5977; CMV-l-glucose, *P* = 0.5392; CMV-O-sucrose, *P* = 0.8677; CMV-l-sucrose, *P* = 0.7759) (Supplementary Fig. 9a–c). In addition to JA, salicylic acid (SA) is another plant hormone involved in stress resistance in plants. To rule out the involvement of these plant hormones in this phenomenon, we further tested aphid wing induction using the *Arabidopsis* mutants, *Δdde2* and *Δsid2*, which cannot synthesize JA and SA, respectively. As a result, there was no significant difference in wing induction among wild type, *Δdde2* and *Δsid2* (*χ*^2^ = 1.672, d.f. = 2, *P* = 0.4335) (Supplementary Fig. 9d). Therefore, we concluded that JA, SA, sucrose and glucose levels were not involved in aphid wing induction (Supplementary Fig. 9). Although we do not believe that the age of virus-infected plants has a significant effect on wing formation, this point still remains to be examined.

We also evaluated aphid population growth and development in opposite conditions with or without population stress. When the intrinsic rate of increase (*r*_m_) of an aphid population (Supplementary Fig. 9e), which can explain how aphid population growth differs, was compared in the absence of population stress, the aphids fed on CMV-infected tobacco have significantly higher values of *r*_m_ than those on healthy or [CMV + Y-sat]-infected plants (ANOVA, Tukey, *P* < 0.0001) (Supplementary Fig. 9f). However, when we compared aphid population size in a high-density condition, the growth of aphid population size was bigger in aphids fed on [CMV + Y-sat]-infected tobacco plants, suggesting that Y-sat can somehow enhance colony growth when aphids are highly populated (ANOVA, Tukey, *P* = 0.0336) (Supplementary Fig. 9g). The effect of Y-sat on aphid colony size seems to be in favour of wing induction.

What are we to conclude from this molecular crosstalk via Y-sat sRNAs that regulate the interactions among the four entities? We believe that our findings demonstrate an extraordinary role for noncoding RNA in viral persistence, especially in establishing an unusual interdependence (possibly a multilateral symbiosis) between a pathogen and its insect vector.

## Methods

### Plants, insect stocks and virus inoculation

Colonies of *M. persicae* (Sulzer) (Insecta: Hemiptera: Aphididae) originally isolated from *N. tabacum* were reared on *Brassica rapa* plants. For different experiments, adults of *M. persicae* were maintained either in a high-density (>20 adults/seedling) or in a low-density (2 adults/seedling) on broad bean (*Vicia faba*) seedlings individually grown in a 50 ml tube with an air-permeable top. Tobacco (*N. tabacum* L. cv BY4) seeds were sown in coir dust (Jiffy-7 pots, Jiffy Products International BV), and seedlings were transplanted to individual pots at the 3–4-leaf stage. Plants and aphids were grown at 24 °C with 16 h of artificial light. CMV-O was used as a helper virus for Y-sat. Tobacco plants were inoculated with purified virions of CMV-O and Y-sat at the 4-leaf stage. An infection led to characteristic yellow symptoms. CMV with Y-sat was maintained in *N. tabacum* plants through successive rub inoculations. CMV-L was also used as a helper virus.

### Life cycle of *M. persicae* with respect to the body colour and wing formation

A single adult aphid was randomly selected from the stock culture and placed on a seedling of *V. faba* in a 50 ml tube (described above). Plants and insects were kept in a climate-controlled incubator at 24 °C with 16 h of artificial light. All experimental data were obtained using viviparous daughters and granddaughters from this adult. All the red and green morphs from a 3-week-old colony were separated and counted. Twenty third-instar green morphs were randomly collected and reared on *V. faba* (five aphids per seedling). All red aphids from the same colony were also collected and reared on *V. faba*. The aphids were observed daily until reproductive maturity, and the fate of each was recorded. The morphology and development, including body colour and wing-bud development, of 25 1-day-old aphids were individually observed daily, and the days until each moult were counted by observing exuviae. A representative green and red morph individual was photographed each day using a Dino-eye (AM321) or Ricoh digital camera (WG-4). The statistical methods used for the analyses of the observations (wing formation, body colony development, intrinsic rate of increase, etc.) are described in detail in each section.

### Detection of *M. persicae* densovirus (MpDNV) in aphids

DNA was extracted from five alate and five apterous aphids at the adult stage using a standard phenol–chlorophorm protocol. MpDNV DNA and complementary DNA (cDNA) were amplified and detected using primer pairs 5′-TGACAATGGGTATATTCATTGACCT-3′/5′-ATCGTGCGTCAAAAGAAACCCT-3′ and 5′-ATCCATGGGCTCCGATGAAT3′/5′GCAAGGGTGTCATGCGTAAT-3′, respectively.

### Aphid attraction bioassay

Winged *M. persicae* aphids from the high-density aphid populations that had fed on *V. faba* were starved for 3 h before the pairwise bioassay. Two *N. tabacum* plants placed 30 cm apart in a 35 × 15 × 20 cm^3^ glass box as shown in Supplementary Fig. 4a. The starved alate aphids (20–30) were placed in a Petri dish in the centre between the two plants. In separate experiments, aphid attraction was compared for CMV-O vs [CMV-O + Y-sat]-infected tobacco plants and for healthy vs [CMV-O + Y-sat]-infected tobacco plants. For 2 h, every 10 min, the number of aphids on each plant was recorded. The experiment was repeated four times. For each replication, the orientation of the plants was changed from left to right and vice versa. For the statistical analysis, the values of all four replicates in each treatment were combined and *χ*^2^ test was performed. The results of the *χ*^2^ test were also confirmed by a hypothetical test for one-sample (two-sample) proportion in R. The aphids that did not show any preference were considered as unimportant data (missing data). Similar experiments were conducted in the dark. The number of the aphids that did not show any preference was also included in the statistical analysis.

### Y-tube bioassay

*N. tabacum* plants infected with CMV-O and with [CMV-O + Y-sat], and virus-free, healthy plants were used. Fifty *M. persicae* aphids were used for each test. As shown in Supplementary Fig. 4b, for each experiment, the test plants were placed in two separate odour source bottles that were connected to the arms of a Y-tube. Air flow was maintained at either 10 or 0.2 L min^−1^ using an air pump fixed to each odour source bottle, measured using flow metres before and after air entered the bottles. One aphid was released at the long arm of the Y-tube and allowed to walk upwind and choose an arm. Once the aphid walked more than halfway into the selected arm, that arm was considered as the aphid’s choice. Each aphid was given a 2 min testing period. After every ten tests, the same plants were placed on different sides of the Y-tube. For the statistical analysis, *χ*^2^ test was conducted. In addition, we also used a hypothesis test for one-sample proportion in R to further ensure the conclusion raised by the *χ*^2^ test. The data sets are all presented in Supplementary Methods 1.

### Variation in wing plasticity in aphids feeding on infected plants and transgenic plants

Twenty 1-day-old aphids (from low-density colonies reared for several generations) were put on healthy *N. tabacum*, CMV-infected *N. tabacum*, [CMV + Y-sat]-infected *N. tabacum*, wild-type *N. benthamiana* and dsY-sat-expressing transgenic *N. benthamiana*. After 28 and 35 days, the alate and the apterous aphids were counted separately. Similarly, alate and apterous morphs in the aphid populations on wild-type or the *A. thaliana* mutants, *Δdde2* (CS6149) or *Δsid2* (CS16438), which were obtained from Arabidopsis Biological Resource Centre in the Ohio State University, were tested. *χ*^2^ test was conducted to know whether there is a statistical difference between the two (alate and apterous) populations.

### CMV quantification in plants

To measure CMV levels in Y-sat-infected *N. tabacum* plants, total RNA was extracted from the plants using a conventional phenol–chloroform method. cDNA was synthesized using Takara Perfect Real Time, PrimeScript^TM^ RT Reagent Kit (Takara, Catalogue# RR037A). Quantitative PCR (qPCR) was performed using SYBR Green Master Mix (Applied Biosystems, Catalogue# A25742) in the StepOnePlus^TM^ Real-Time PCR System (Applied Biosystems). The 18S ribosomal RNA gene was used as the reference gene; primer pair 5′-GCAAGACCGAAACTCAAGG-3′/5′-TGTTCATATGTCAAGGGCTGG-3′ was used. For CMV detection, primer pair 5′-GTTGACGTCGAGCACCAACGC-3′/5′-TGGTCTCCTTTTGGAGGCCC-3′ was used. Real-time qPCR was performed and the data were processed as explained in Supplementary Note 3. To detect a difference in CMV accumulation level in infected plants between the two samples, two-tailed *t* test was performed. The data sets are presented in Supplementary Methods 1.

### CMV quantification in individual aphids

Aphids were maintained on *B. rapa* plants. Apterous aphids were starved for 3 h and then allowed a 2–5-min virus acquisition access period on CMV-infected or [CMV + Y-sat]-infected *N. tabacum* 10–12 d.p.i. The aphids were collected and placed in separate 2-ml screw-cap tubes with two beads (Ø4.5 mm), then homogenized with 500 µl Trizol (Invitrogen, Catalogue# 15596018) using a Tomy Micro Smash MS-100 (Digital Biology) at 4000 rpm for 30 s. Total RNA was extracted by adding 100 µl of chloroform followed by centrifugation and RNA precipitation. Total RNA was recovered by centrifugation and dissolved in 6 µl of RNase-free water and reverse-transcribed using AMV reverse transcriptase (Nippon gene, Catalogue# 311-07501). Quantitative PCR was performed as described above. The partial ribosomal protein S3 (*Rps3*) gene from *M. persicae* was used as the reference gene and amplified by primer pair 5′-CGAGCTGCTCCATCTCGTA-3′/5′-CCCACTTTCCATGATGAATCTCA-3′, which was designed by Kim et al.^31^. The primer pair for CMV was described above. Real-time qPCR was performed and the data were processed as explained in Supplementary Note 3. To detect a difference in CMV accumulation level in aphids between the two samples, two-tailed *t* test was performed. The data sets are presented in Supplementary Methods 1.

### Quantification of *Apns1-like* mRNA in aphids

One-day-old aphids (20 from the low-density colonies reared for several generations) were placed on separate transgenic *N. benthamiana* plants expressing Y-sat dsRNA or on wild-type *N. benthamiana* plants. Total RNA was extracted from ten aphids at the third instar and from a single aphid at the adult stage. *Apns1-like* mRNA was amplified by primer pair 5′-CCGGCGCAATGACGGAACTGA-3′/5′-CCATGGCACCAACGGCGATGA-3′. The β-tubulin gene from *M. persicae* was used as the reference gene and amplified by primer pair 5′-CCATCTAGTGTCGCTGACCA3′/5′-GTTCTTGGCGTCGAACATTT-3′, which was previously described by Kang et al.^32^. Real-time qPCR was performed and the data were processed as explained in Supplementary Note 3. To detect a difference in *Apns1-like* mRNA accumulation level in aphids, ANOVA followed by Tukey’s test was performed. The data sets are presented in Supplementary Methods 1.

### Quantification of *ABCG4* in aphids

Aphids were put on CMV-infected *N. tabacum*, [CMV-O + Y-sat]-infected *N. tabacum*, transgenic *N. benthamiana* expressing Y-sat dsRNA and wild-type *N. benthamiana*. For aphid *ABCG4* mRNA (XM_022308170.1), total RNA was extracted separately from alate aphids of the third instar and from alate adults and amplified using primer pair 5′-AACTGCCCTGTCCCATCTAT-3′/5′-GGTGTGTCATTGATGGCTAG-3′. The *β-tubulin* gene was used as the reference as described above. Real-time qPCR was performed and the data were processed as explained in Supplementary Note 3. To detect a difference in *ABCG4* mRNA accumulation level in aphids, two-tailed *t* test was performed. The data sets are presented in Supplementary Methods 1.

### RNA dot-blot hybridization

Synthetic RNA oligonucleotides of Y-sat RNA (5′-ACGAUGGUGGGAGUCACCCAAGGGG-3′), *ABCG4* RNA (5′-AAUUGGAAAAGGAACCAAAGA-3′) and miR171b (5′-UGAUUGAGCCGCGCCAAUAUC-3′) as the negative control were dot-blotted in a dilution series (10, 4, 1 and 0.4 pmol) onto an Amersham Hybond-N + membrane (GE Healthcare, Catalogue# RPN303 N). The membrane was soaked in 20× SSC buffer (3 M NaCl, 0.3 M sodium citrate) and cross-linked using UV Crosslinker CX-2000 (UVP, Catalogue# 3-3387-01). The dotted RNAs were probed with 50 nM digoxigenin (DIG)-tagged miR9b (miR9b, 5′-UCUUUGGUACUUUAGCUGUAG-3′) in the hybridization buffer (50% formamide, 0.63 M NaCl, 60 mM sodium citrate, 20 mM maleic acid, 2% blocking reagent [Roche, Catalogue# 11096176001], 7% SDS, 0.6% *N*-lauryl sarcosine, 25 mM Na_2_HPO_4_) at 40 °C overnight. After a wash with the washing buffer (0.3 M NaCl, 30 mM sodium citrate, 0.1% SDS) and then blocking in the blocking buffer (0.1 M maleic acid, 0.15 M NaCl, 1% Blocking reagent) for 1 h, the membrane was treated with anti-DIG-AP, Fab fragments (Sigma-Aldrich, Catalogue# 11093274910) in the DIG reaction buffer (0.1 M maleic acid, 0.15 M NaCl, 1% Blocking reagent, 0.3% Tween-20) for 0.5 h. After washing with the DIG washing buffer (0.1 M maleic acid, 0.15 M NaCl and 0.3% Tween-20), the CDP-star substrate (Roche, 11759051001) was added, and luminescence in the alkaline phosphatase reaction was visualized using the LAS4000 Luminescent Image Analyzer (Fujifilm).

### Competitive hybridization assay

The probe miR9b (100 pmole) was first preincubated at room temperature for 1 h with Y-sat RNA at molar ratios of 0, 0.5 and 1.0 after the incubation at 65 °C for 5 min. miR9b with Y-sat RNA was then hybridized with dot-blotted *ABCG4* RNA as described above.

### Aphid transmission test

*M. persicae* individuals were raised on healthy *B. rapa*, then apterous aphids were starved for 3 h, and allowed a 2–5-min acquisition access period on CMV- or [CMV-O + Y-sat]-infected *N. tabacum* at 10–12 d.p.i. Aphids were confirmed to have probed on a leaf, then ten aphids were collected and transferred to a healthy *N. tabacum* plant using a fine, dry paintbrush. The aphids were then killed with a pesticide after 1 day. Infection of plants was confirmed by visual symptoms after 1–2 weeks. Ten test plants were used for each transmission experiment, and the experiment was done two or three times. Similar experiments were conducted for the viral transmission efficiency of apterous and alate aphids (A1). Sap from [CMV-O + Y-sat]-infected *N. tabacum* was used as an inoculum. To test whether there is a statistically significant difference between [CMV + Y-sat] and CMV (or Healthy), we used *χ*^2^ test. For the data set, please see Supplementary Methods 1.

### Aphid intrinsic rate of increase

One aphid per clip cage, two cages per plant and 30 plants per infection type were used (Supplementary Fig. 9e). Newly emerged apterous aphids (<1-day-old) were confined in a clip cage on the fully expanded 4th or 5th leaf of 8-week-old *N. tabacum* plants (healthy, CMV-infected and [CMV-O + Y-sat]-infected, 10–15 d.p.i.) for 7 days. The intrinsic rate of increase (*r*_m_) for each aphid was calculated as *r*_m_ = 0.738(Log_e_ *N*)/*d* as proposed by Wyatt and White^33^, where *d* is the number of days to the first generation of offspring and *N* is the number of nymphs produced by each aphid. Depending on the survival, *r*_m_ was calculated from 36, 52 and 42 individuals for healthy, CMV-infected or [CMV-O + Y-sat]-infected *N. tabacum*, respectively. To detect a difference in intrinsic rate of increase among the three samples (Healthy, CMV-O and [CMV-O + Y-sat]), ANOVA followed by Tukey’s test was performed. The data sets are presented in Supplementary Methods 1.

### JA analysis

Levels of JA in the leaves from four plants each of healthy, CMV-infected and [CMV-O + Y-sat]-infected *N. tabacum* at 45 d.p.i. were measured. Samples were weighed, frozen in liquid nitrogen and stored at −80 °C until used for ultra-performance liquid chromatography-tandem mass spectrometry as described previously^34^. To know whether there is a difference among the three samples (Healthy, CMV-O and [CMV-O + Y-sat]), ANOVA followed by Tukey’s test was performed. The data sets are presented in Supplementary Methods 1.

### Phloem sap analysis

Sucrose and glucose levels were measured in leaves from three plants each of healthy, CMV-infected and [CMV + Y-sat]-infected *N. tabacum* at 45 d.p.i. Leaf tissue (0.1–0.2 g) was frozen in liquid nitrogen and ground using a sterilized mortar and pestle, then transferred to 1.5 ml tubes with two volumes of 80% ethanol, and incubated at 40 °C for 18 h. The supernatant after centrifugation at 9000 × *g* for 10 min was transferred to clean 1.5 ml tubes, vacuum-evaporated completely and centrifuged at 3000 × *g* for 10 min. The supernatant was transferred to new 1.5 ml tubes and stored at −20 °C until analysis. Sucrose and glucose levels in the samples were analysed using Boehringer Mannheim Enzymatic BioAnalysis for sucrose, d-glucose and d-fructose (R-Biopharm AG, Catalogue# 0-139-041) and the manufacturer’s protocol. To know whether there is a difference in sugar level among the three samples (Healthy, CMV-O and [CMV-O + Y-sat]), ANOVA followed by Tukey’s test was performed. The data sets are presented in Supplementary Methods 1.

### Photosynthetic rate

Five-week-old plants of *N. tabacum*, grown in a plant growth room with a 15 h light/9 h dark at 24 °C, were rub-inoculated with either CMV-L, [CMV-L + Y-sat], CMV-O or [CMV-O- + Y-sat]. The photosynthetic rate was measured for three individual plants, twice for each plant at 15 and at 30 d.p.i. using a miniPPM-200 (EARS Plant Photosynthesis Monitoring BV, Kanaalweg) and the manufacturer’s protocol. Plants were first dark-adapted in the dark overnight. Initially, the *F*_ref_ (reference fluorescence) value was fixed by doing a pre-test in the dark (PPFD = 0 µmol m^−2^ s^−1^). The first measurement was done in the dark condition (PPFD = 0 µmol m^−2^ s^−^), and light intensity was increased gradually, allowing a 10–15-min adaptation before the measurements. To know whether there is a difference in photosynthetic rate among the three samples (Healthy, CMV-O and [CMV-O + Y-sat]), ANOVA followed by Tukey’s test was performed. The data sets are presented in Supplementary Methods 1.

### Stomata conductance

Using the same setup and plants used to measure photosynthesis, stomata conductance was measured using an SC-1 Leaf Porometer (Decagon Devices) and the manufacturer’s instructions. Measurements were done from 11.00 a.m. to 3.00 p.m. at 24–27 °C. To know whether there is a difference in stomata conductance among the three samples (Healthy, CMV-O and [CMV-O + Y-sat]), ANOVA followed by Tukey’s test was performed. The data sets are presented in Supplementary Methods 1.

### Wing induction study on plants transiently expressing Y-sat

Thirty-five 1-day-old aphids (from low-density colonies reared for several generations) were put on *N. benthamiana* leaves transiently expressing a Y-sat sequence (plus-sense, negative-sense or dsRNA) 1 day after agroinfiltration. Four plants were used for replicates (*n* = 4). The alate aphids and the apterous aphids were counted separately 4 days later. For the transient expression, the Y-sat sequences were inserted into pBE2113 Ti plasmid. The recombinant pBE2113 vectors were introduced into *Agrobacterium tumefaciens* using a conventional freeze–thaw method and cultured in YEP medium (1% yeast extract, 1% peptone, 0.5% NaCl and 50 µl ml^−1^ kanamycin) and incubated 48 h. Bacterial cells were pelleted by centrifugation and suspended in agrobacterium resuspension buffer (10 mM MgCl_2_, 10 mM MES and 0.2 mM acetosyringone), adjusted to 0.6 at OD_600_ and allowed to sit for 2–3 h. Leaves were then infiltrated with the inoculum. The *GUS* gene was used as the control (Supplementary Fig. 10a). To know whether there is a difference in aphid wing induction between the control (GUS) and each sample (Y-sat+, Y-sat- or dsY-sat), ANOVA followed by Dunnett’s test was performed. The data sets are presented in Supplementary Methods 1.

### Wing induction study on Y-sat-transgenic plants

Thirty aphids (from low-density colonies reared for several generations) were put on transgenic *N. benthamiana* plants expressing a Y-sat sequence (plus-sense, negative-sense or dsRNA). Four plants were used for replicates (*n* = 4). Nymphs of alate aphids and apterous aphids were separately counted by red and green morphs 5 days after aphid introduction. To know whether there is a difference in aphid wing induction among the four samples (dsY-sat, Y-sat+, Y-sat- and Wild), ANOVA followed by Tukey’s test was performed. The data sets are presented in Supplementary Methods 1.

### Exogenous Application of Y-sat dsRNA into aphids through artificial feeding

A standard artificial basal diet previously developed for *M. persicae* was supplemented with Y-sat dsRNA or leek yellow stripe virus (LYSV) (dsRNA control). The artificial diet consisted of amino acids, vitamins, K_3_PO_4_, MgCl_2_ and sucrose as previously reported^35^. The pH of the diet was adjusted to 7.0 before filter sterilization. The feeding apparatus (Supplementary Fig. 10b) was described previously^36^. The feeding apparatus was constructed using Petri dish of 3.5 cm in diameter and with a lid of 4 cm in diameter. The food sachet was made by slightly depressing a piece of Parafilm membrane, on top of the Petri dish. The artificial diet (200 µl) was then pipetted on the membrane and 7 µl of dsRNA (700 ng) or dH_2_O was mixed by pipetting. The diet was then covered with another piece of Parafilm that had been stretched four times.

Thirty aphids (2–3 days old) from the low-density colonies, which had been fed on healthy *B. rapa* plants for several generations, were carefully placed on the lid using a camel brush. The Petri dish was then placed on the lid and a Parafilm ring (see Supplementary Fig. 10b) was placed inside of the lid to maintain the inner gap between the lid and the Petri dish. A hole (Ø = 1 cm) was made on the lid that was covered with a net for ventilation. Nymphs of the alate and the apterous aphids were counted after 4 days. Negative controls are the sample without any dsRNA and with LYSV dsRNA, whose sequence is unrelated to that of Y-sat. To know whether there is a difference in aphid wing induction among the three samples (Control, dsY-sat and LYSV), ANOVA followed by Tukey’s test was performed. The data sets are presented in Supplementary Methods 1.

### sRNA-seq analysis

Total RNA was extracted from a bulked sample of aphids that had fed on CMV- or [CMV + Y-sat]-infected *N. benthamiana* plants. RNA samples were sent to Macrogen (Korea) for sRNA-seq. The raw data were first quality-checked by Sickle (ver. 1.33) (https://github.com/najoshi/sickle), siRNAs (18–35 nt) were then extracted and used to analyse the size-specific distribution of sRNAs. sRNAs derived from the aphid genome, CMV and Y-sat RNAs were sorted by SAMtools (ver. 1.2) (https://sourceforge.net/projects/samtools/files/samtools/1.2/). The aligned reads were extracted to the bam2fastq format (www.htslib.org). Size-specific distribution of the aligned reads was analysed using the programming language C in Microsoft Visual Studio 2019 (ver. 16.4.2).

### Statistical analyses

No statistical methods were used to predetermine the sample sizes. Experimental groups were randomized. Investigators were not blinded during experiments and outcome assessment. Data are shown as individual data points with means ± SE. The numbers of the samples used in each experiment are annotated as ‘*n*’. Statistical analyses were done using the software GraphPad Prism (GraphPad ver. 8.4.3) and R 3.6.3. Normally distributed data were analysed using either unpaired two-tailed *t* tests for single comparisons, one-way ANOVAs for multiple comparisons or two-way ANOVAs for comparison of groups over time. When the sample sizes were different among the replicates (Fig. 4j), GLM in R was used. Tukey’s or Dunnett’s test was used for mean separation. For the contingency analysis, *χ*^2^ test was conducted. In addition, to ensure the results of the contingency analysis, a hypothesis test for two-sample proportion in R was performed. *P* < 0.05 was considered to indicate statistical significance; *P < 0.05, **P < 0.01, ***P < 0.001 and ****P < 0.0001.

### Reporting summary

Further information on research design is available in the Nature Research Reporting Summary linked to this article.

## Supplementary information

Supplementary Information

Reporting Summary

## Data Availability

All data generated or analysed in this study, which support the findings of this study, are included in this published article and Source data. The raw sRNA-sequencing data used in this study are available in the DNA Data Bank of Japan (DDBJ) database under the DDBJ Sequence Read Archive (DRA) accession code DRA013038. Source data are provided with this paper.

## Source data

Source Data

## Code availability

A code for the analysis of small RNA size distribution in language C is available in Supplementary Methods 2.
