# Supplementary material for: A plant virus satellite RNA directly accelerates wing formation in its insect vector for spread
Source: Nat Commun. 2021 Dec 6;12:7087. doi: 10.1038/s41467-021-27330-4 (PMC8648847; doi:10.1038/s41467-021-27330-4)
Supplement: Supplementary file 1 — Supplementary Information [file 41467_2021_27330_MOESM1_ESM.pdf]

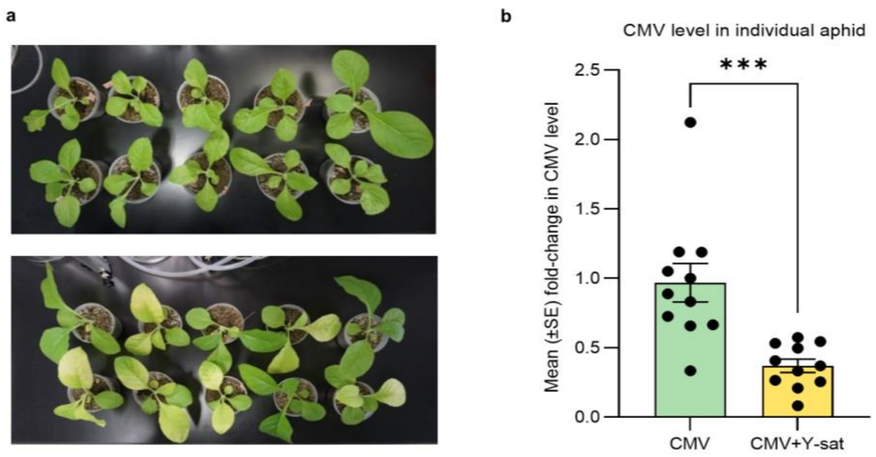

Supplementary Fig. 1 Aphid transmission of CMV and CMV+Y-sat is not correlated to virus titre in the plant. Virus titre in the Y-sat-infected plants is shown in Fig. 1. **a,** Viral symptoms on *N. tabacum* plants inoculated by aphids (Methods). Green mosaic symptoms on CMV-O-infected *N. tabacum* (a, upper). Yellow symptoms on [CMV-O+Y-sat]-infected *N. tabacum* (a, lower). **b,** CMV levels in individual aphids, which had fed on CMV-infected or [CMV+Y-sat]-infected plants, were measured by qPCR. Aphids on CMV-infected plants contained 2.6 times more CMV than the aphids fed on [CMV+Y-sat]-infected plants (two-sided *t*-test, n = 11, P = 0.0006). ***P < 0.001. Black dots represent individual data points (relative CMV levels of individual aphids). Source data are provided as a Source Data file.

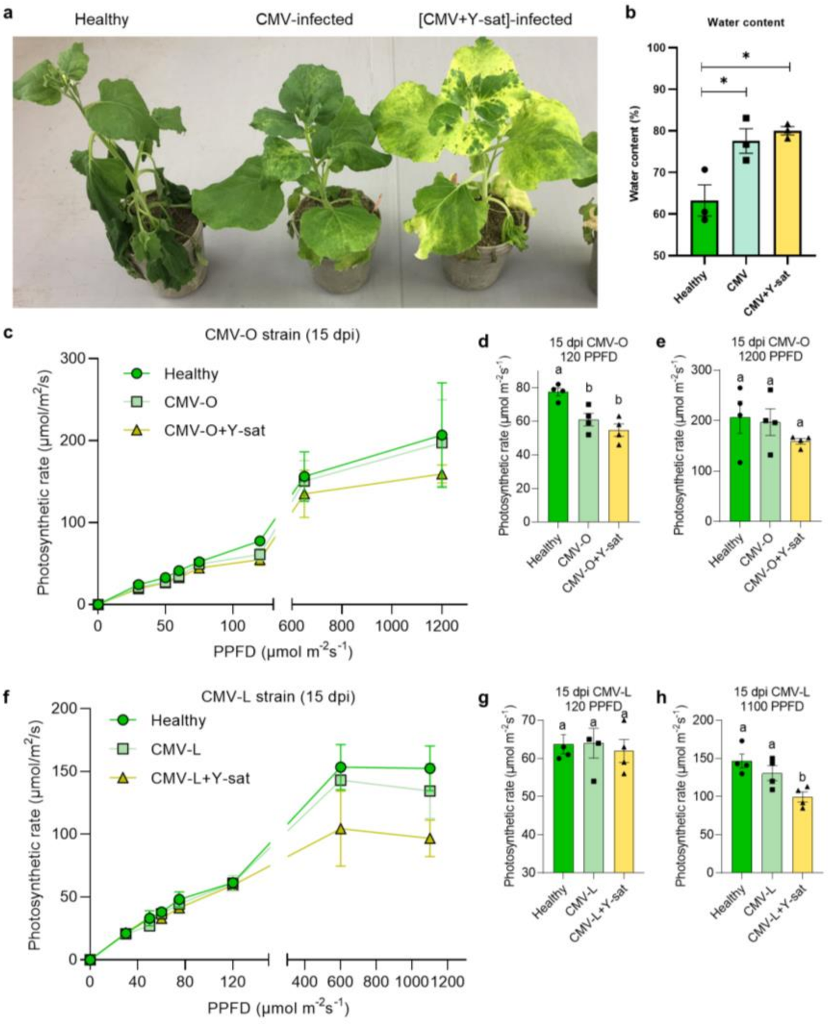

Supplementary Fig. 2. Drought stress symptoms and photosynthetic rates in healthy and infected plants a, Effect of drought stress on healthy, CMV-infected and [CMV+Y-sat]-infected *N. benthamiana* after seven days without watering. **b,** Water content (WC) of each plant was indicated in percentage by the following calculation: WC = [(fresh weight — dry weight)/fresh weight]x100%. Healthy, CMV-infected and [CMV+Y-sat]-infected *N. benthamiana* plants were compared (one-way ANOVA, Tukey's test, n = 11, P = 0.0115). **c-h,** Mean (±SE) photosynthetic rates in healthy and infected plants at 15 days after inoculation (dpi). Individual data points are mean values of each plant and four plants were used for each treatment**. c and f,** Change in rate in tobacco plants infected with CMV-O (**c,** n = 4) or CMV-L (**f,** n = 3) in response to light intensity (μmolm^{-2}s^{-1} photosynthetic photon flux density,

PPFD). **d, e, g, h,** Mean (±SE) photosynthetic rate among infection types were analysed at 120 PPFD and 1200 PPFD by one-way ANOVA with Tukey's multiple comparison test (n = 4, values followed by the same letters are not significantly different at $P < 0.05$). **d,** CMV-O-infected, 120 PPFD ($P = 0.003$), **e,** CMV-O-infected, 1200 PPFD ($P = 0.3769$). **g,** CMV-L-infected, 120 PPFD ($P = 0.8239$), **h,** CMV-L-infected, 1100 PPFD ($P = 0.0259$). Source data are provided as a Source Data file.

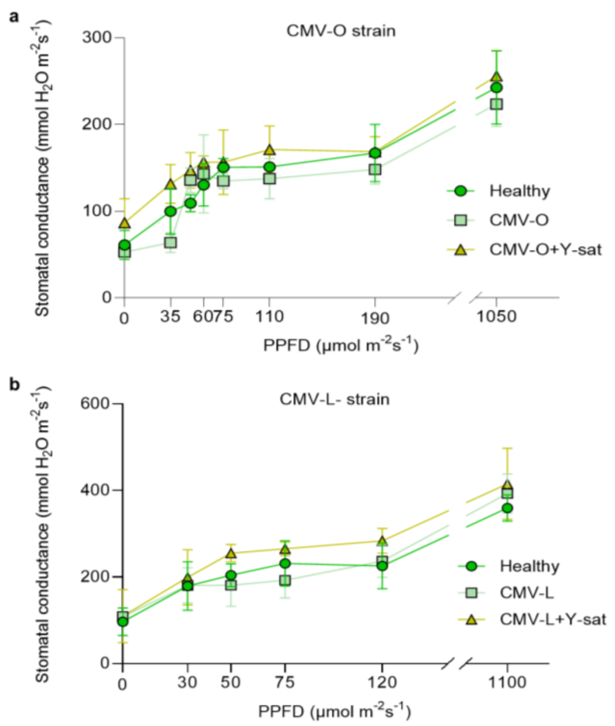

Supplementary Fig. 3 Stomatal conductance of healthy and infected plants in response to light intensity at 30 days after inoculation (dpi). Symptoms of CMV-infected and [CMV+Y-sat]-infected plants became much milder due to host silencing at 30 dpi than at 15 dpi but [CMV+Y-sat]-infected plants still had more red aphids than on the CMV-infected. Two CMV strains (CMV-O and CMV-L) were used. **a,** Mean (±SE) stomatal conductance of CMV-O-infected, [CMV-O+Y-sat]-infected and healthy tobacco plants. (n = 3). **b,** Mean (±SE) stomatal conductance of CMV-L-infected, [CMV-L+Y-sat]-infected and healthy tobacco plants (n = 3). Individual data points are mean values of each plant. Source data are provided as a Source Data file.

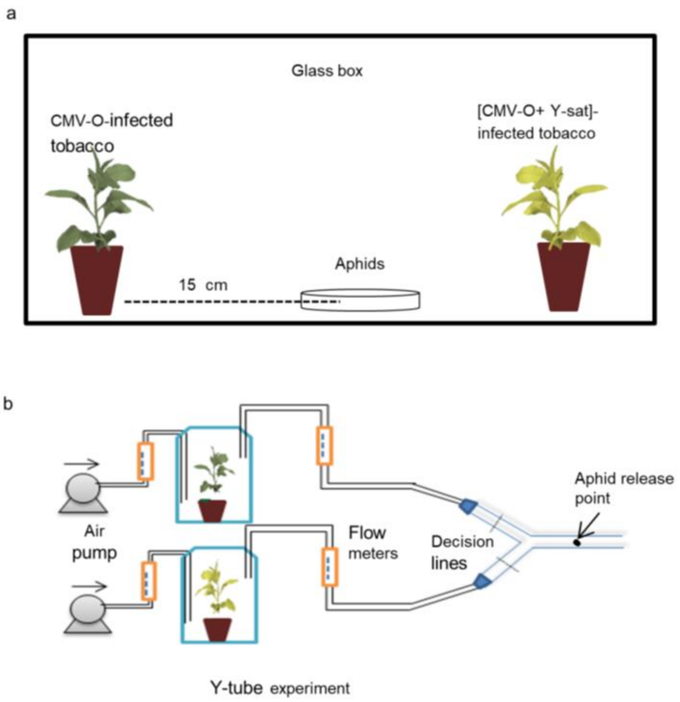

Supplementary Fig. 4 Experimental designs for aphid bioassays (Methods). a, Aphid attraction bioassay. **b,** Y-tube aphid attraction bioassay. See methods for more details.

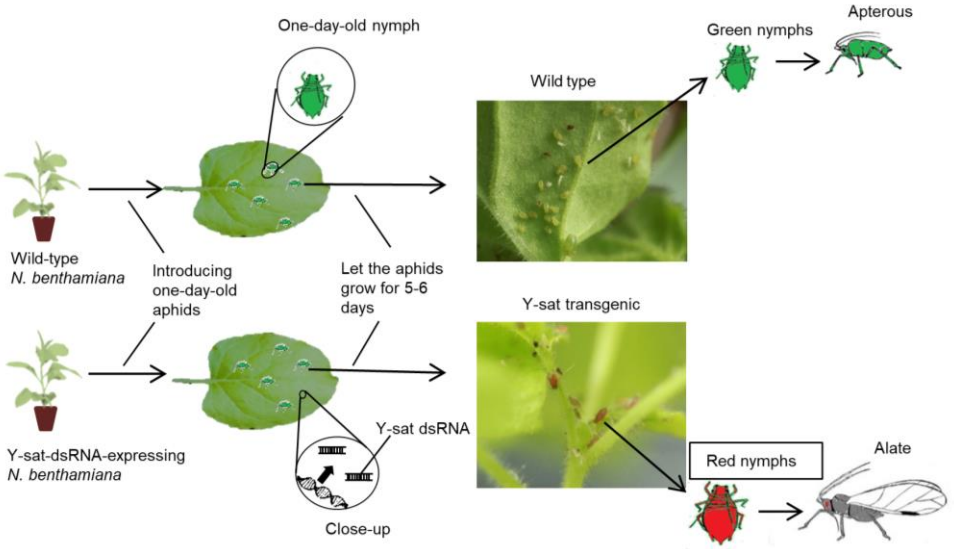

Supplementary Fig. 5 Schematic representation of Y-sat-mediated metamorphosis to alate aphids. Y-sat can accelerate aphid wing formation. Aphids that fed on transgenic plants expressing Y-sat dsRNA frequently turned red and eventually changed into alates.

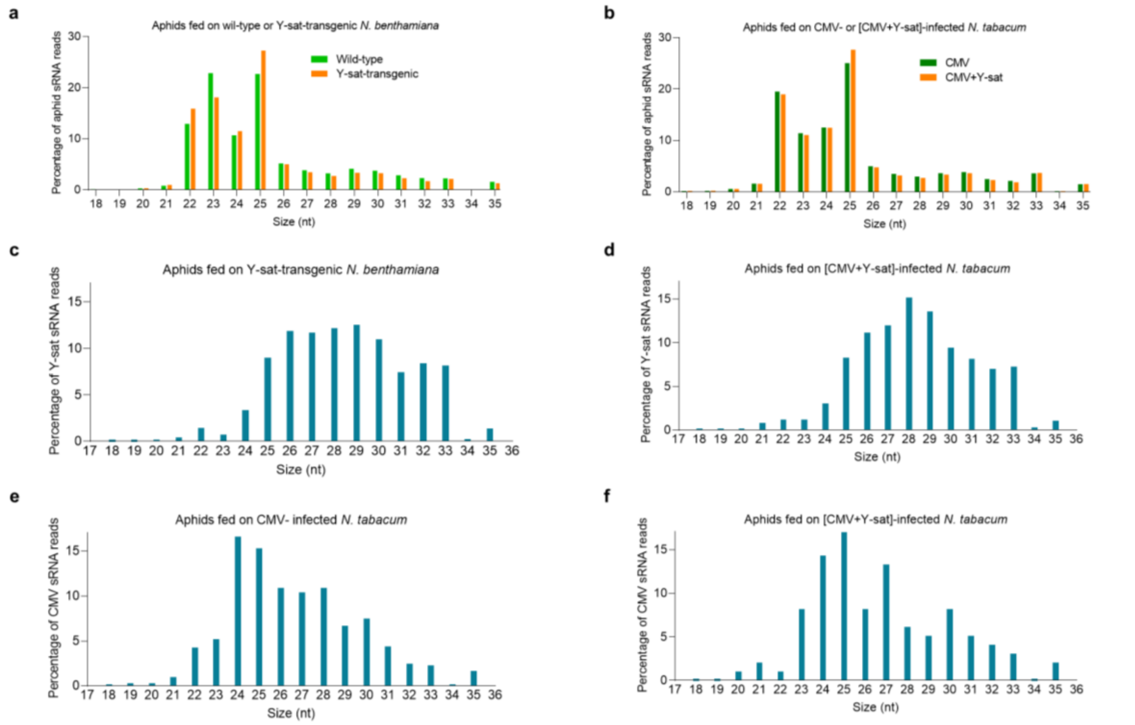

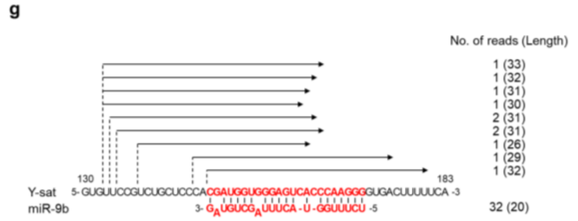

h

Sample	No. of paired reads	High-quality paired read count (18-35 bases)	Types of miRs detected in miRDeep2
Aphids from CMV	60,921,079	15,778,386	76
Aphids from [CMV+Y-sat]	83,913,396	20,446,095	86

Supplementary Fig. 6 Piwi-interacting RNAs (piRNAs) derived from Y-sat dsRNA. Size distribution of aphid sRNA reads generated in aphids that fed on **a,** wild-type *N. benthamiana* or transgenic plants expressing Y-sat dsRNA and on **b,** CMV-infected or [CMV+Y-sat]-infected tobacco plants. Size distribution of Y-sat sRNA reads generated in aphids that fed on **c,** transgenic *N. benthamiana* plants expressing Y-sat dsRNA, on **d**, [CMV+Y-sat]-infected tobacco, on **e**, CMV-infected tobacco and on **f,** [CMV+Y-sat]-infected tobacco plants. Peaks of Y-sat sRNAs generated in aphids were around 25-33 nt; those of CMV sRNAs were shorter (24-28nt). **g,** Y-sat reads that show some complementarity to miR-9b. Number of mature miR-9b reads and Y-sat reads complementary to miR-9b were 32 and 11, respectively; total number of reads mapped to the Y-sat sequence was 1960. **h,** Summary of the next-generation sequencing read counts.

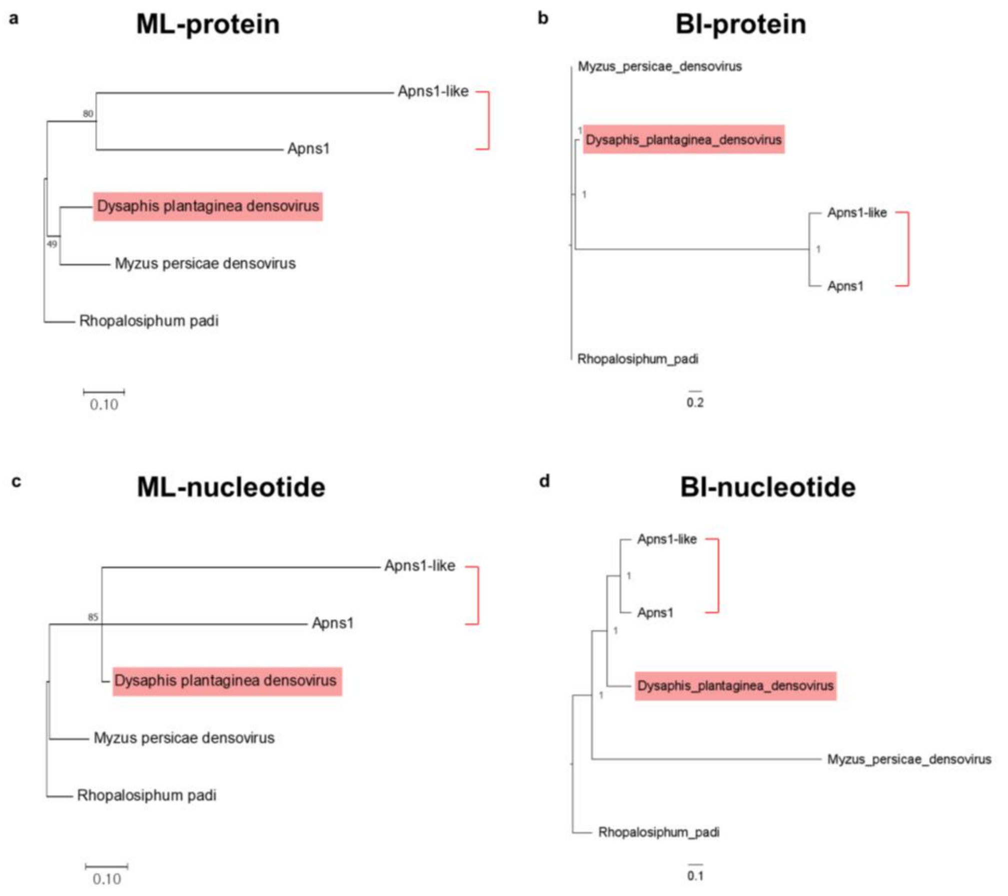

Supplementary Fig. 7 Phylogenetic analysis for the *Myzus Apns1-like* gene in relation to the *Apns1* gene. The amino acid sequences of the densovirus-related proteins and the nucleotide sequences of the corresponding genes were aligned using Clustal W. Phylogenetic analyses were conducted using MEGA7 for Maximum Likelihood (ML) and BEAST v1.10.4 for Bayesian phylogenetic tree interface (BI). **a,** Protein phylogenetic tree was constructed on the JTT model using ML. The bootstrap values from 1,000 replicates are indicated at each node. **b,** Protein phylogenetic tree was constructed on the same JTT model using BI. The posterior probabilities are indicated at each node from 10,000,000 Monte-Carlo Markov Chain (MCMC) length. **c,** Nucleotide phylogenetic tree was constructed using the ML method on the Hasegawa-Kishino-Yano (HKY) model. The bootstrap values from 1,000 replicates are indicated at each node. **d,** Nucleotide phylogenetic tree was constructed using BI on the same HKY model. The posterior probabilities from 10,000,000 MCMC length are

shown at each node. For all four trees, all the positions containing gapes and missing data were eliminated. The branch lengths are measured in the number of substitutions per site. The outgroup is set to the sequence of *Rhopalosiphum padi.* Note that the *Apns1-like* gene was placed next to *Apns1* (red right angle bracket) and closer to Dysaphis plantaginea densovirus (highlighted) than to Myzus persicase densovirus. The gene IDs of Aphidbase or Genbank accession numbers are *Apns1* (ACYPI085607 [https://bipaa.genouest.org/sp/acyrthosiphon_pisum/feature/Acyrthosiphon/pisum/mRNA/ACYPI085607-RA]), *Apns1-like* (MYZPE13164_0_v1.0_000125320.4 [https://bipaa.genouest.org/sp/myzus_persicae/feature/Myzus/persicae/mRNA/MYZPE13164_0_v1.0_000125320.4]), Dysaphis plantaginea densovirus (ACG50803.1 [https://www.ncbi.nlm.nih.gov/protein/ACG50803.1]), Myzus persicae densovirus (NP_874376.1 [https://www.ncbi.nlm.nih.gov/protein/NP_874376.1/]) and *Rhopalosiphum padi* (Rpa20011.t1 [https://bipaa.genouest.org/sp/rhopalosiphum_padi/feature/Rhopalosiphum/padi/mRNA/Rpa20011.t1]). The two homolog genes, *Apns1* and *Apns1-like* are linked by red lines.

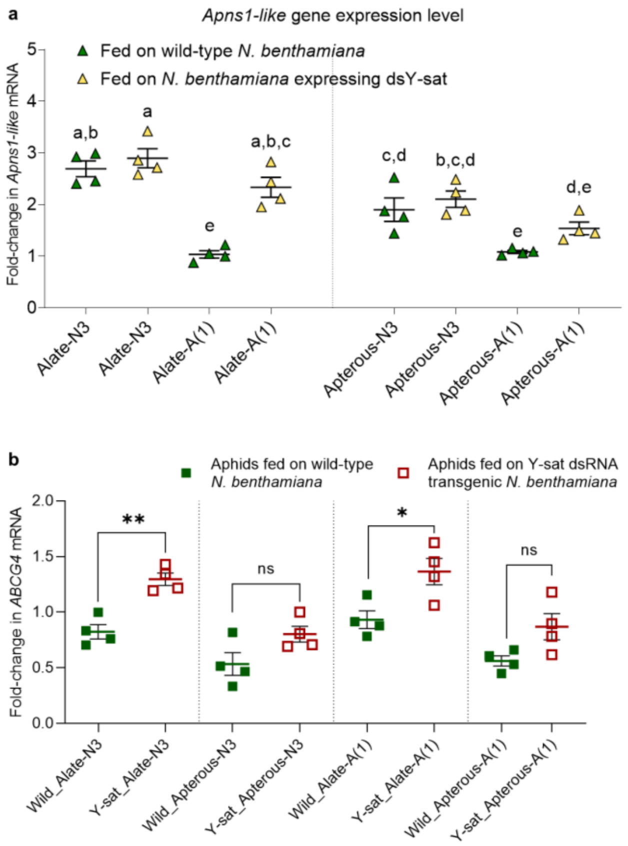

Supplementary Fig. 8 Fold-change in relative expression of *Apns1-like* and *ABCG4* mRNA between aphids at different developmental stages. a, Relative expression levels of *Apns1-like* mRNA in aphids fed on CMV-infected or [CMV+Y-sat]-infected plants. 3rd instars (N3) and early adult [A(1)] are as explained in Fig. 2a (one-way ANOVA, Tukey's test, $n = 4$, $P < 0.0001$). **b,** *ABCG4* mRNA in N3 and A(1) (4-5 days after the N3 stage) of alate and apterous morphs fed on wild-type and transgenic *N. benthamiana* plants expressing Y-sat dsRNA were measured by qPCR. Values are presented as a fold-change. Expression of *ABCG4* was significantly higher in alate aphids fed on transgenic plants at all developmental stages [two-sided *t*-test, $n = 4$, alate N3, $P = 0.0014$; alate A(1), $P = 0.0029$]. There was little significant difference in apterous aphids (two tailed *t*- test). Data are means ± SE. ** $P < 0.01$, * $P < 0.05$. Source data are provided as a Source Data file.

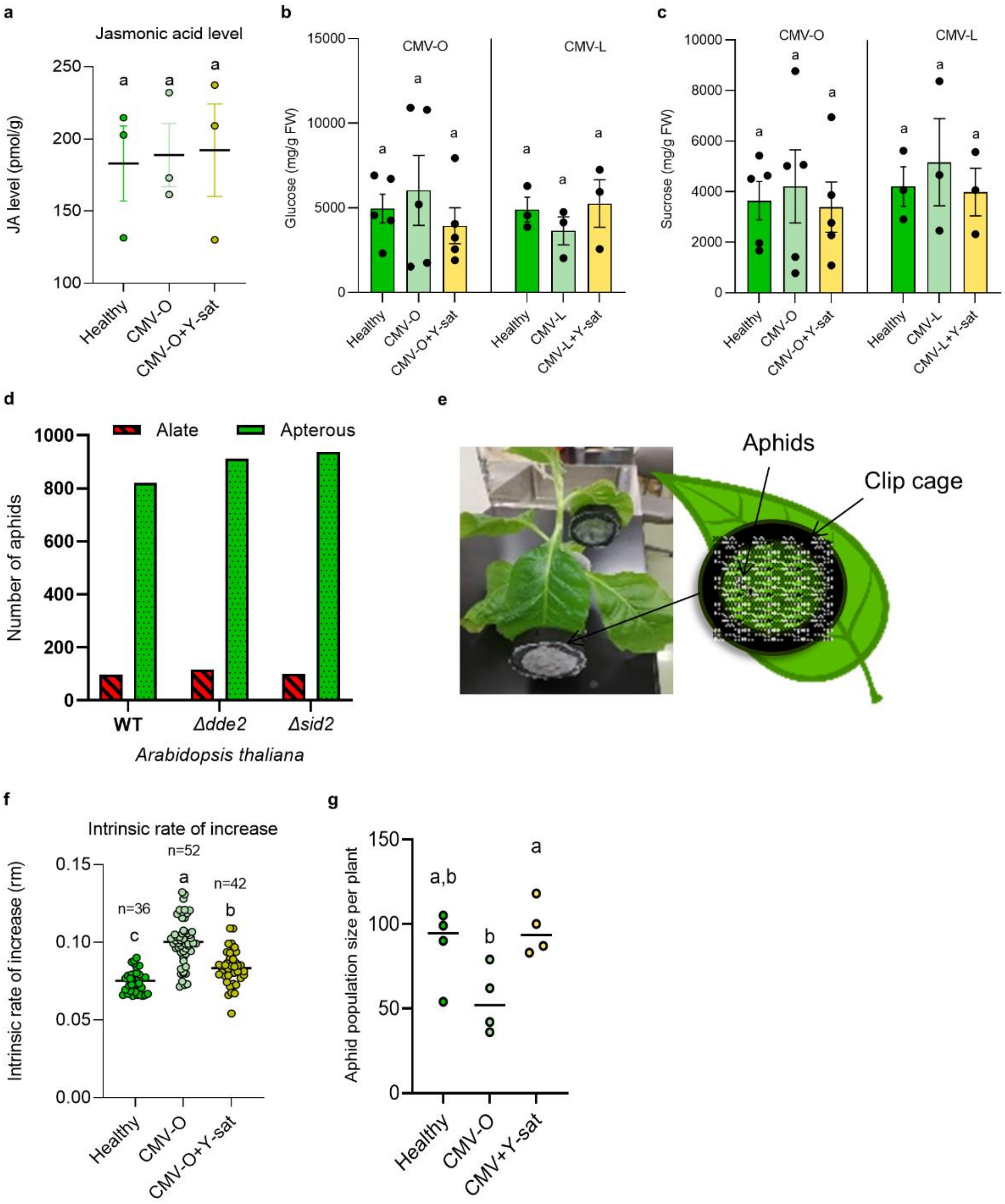

Supplementary Fig. 9 Jasmonic acid levels, phloem sap quality and aphid growth and reproduction. We measured jasmonic acid (JA) levels in leaf tissues at 45 days after inoculation (dpi), when the viral symptoms had become attenuated by host silencing and viral and Y-sat levels were at a steady state, because in the initial stage of infection, levels of plant hormones and sugars varied greatly depending on the individual plant. More alate aphids were still present on [CMV+Y-sat]-infected plants at 45 dpi than on the other samples. **a,** JA levels in healthy, CMV-infected and [CMV+Y-sat]-infected tobacco plants. JA levels in upper leaves did not differ significantly at 45 dpi post-inoculation; JA levels in healthy,

CMV-infected and [CMV+Y-sat]-infected plants were 182.9, 188.8 and 192.1 pmol g^{-1}, respectively. JA levels were not significantly different among the three plants (ANOVA, P = 0.9707). Individual data points represent the JA levels in each plant. **b,** D-glucose level in healthy, CMV-infected and [CMV+Y-sat]-infected tobacco leaves. Two CMV strains (CMV-O and CMV-L) were used. Mean (±SE) D-glucose level did not differ significantly among the CMV-O-infected (6.039 mg gFW^{-1}), Y-sat-infected (3.94 mg gFW^{-1}) and healthy leaves (4.957 mg gFW^{-1}) (ANOVA, P = 0.5977). Similar results were obtained when CMV-L was used. Mean (±SE) D-glucose level in CMV-L-infected, [CMV-L+Y-sat]-infected and healthy leaves was 3.643, 5.255 and 4.908 mg gFW^{-1}, respectively (ANOVA, P = 0.5392). **c,** Sucrose level in the plants used in **b**. Mean (±SE) sucrose level in CMV-O-infected (4.207 mg gFW^{-1}), [CMV-O+Y-sat]-infected (3.389 mg gFW^{-1}) and healthy leaves (3.645 mg gFW^{-1}) did not differ significantly (ANOVA, P = 0.8677). Similar results were obtained when CMV-L was used. Mean (±SE) sucrose level in CMV-L-infected, [CMV-L+Y-sat]-infected and healthy leaves was 5.166, 3.986 and 4.203 mg gFW^{-1}, respectively (ANOVA, P = 0.7759). Each data point is the glucose and sucrose levels in each plant. The data in **a**-**c** were analyzed using ANOVA followed by Tukey's test and values followed by the same letter were not significantly different in Tukey's test at 95% confidence. **d,** Mean percentages of alate and apterous morphs of aphid populations infesting wild-type or transgenic *A. thaliana* plants *Δdde2* or *Δsid2* (chi-squared test, P = 0.4335). Forty of one-day-old aphids were placed on each plant and the proportion of alate or apterous aphids were compared at 14 days post-infestation. **e,** Schematic representation of experimental design for attachment of clip-cages to *N. tabacum* plant used to measure r_m (Methods). **f,** Intrinsic rate of increase (r_m) of aphids reared on CMV-infected, [CMV+Y-sat]-infected and healthy plants. The r_m of aphids fed on [CMV+Y-sat]-infected plants was significantly lower than that of CMV-infected plants, but did not differ significantly compared to the healthy. Values followed by the same letters are

not significantly different. Each data point is r_m of each aphid (ANOVA, Tukey's test, P < 0.0001). **g,** Mean size of aphid population on healthy, CMV- infected and [CMV+Y-sat]-infected *N. tabacum* (ANOVA, Tukey's test, P = 0.0336). Each data point represents the sizes of aphid population on each tested plant. Twenty of one-day-old aphids were placed on each plant and the total number of offspring aphids were counted after 10 days. Note that in a low-density condition, the intrinsic rate of individual aphid was a little higher in the aphids on CMV-infected plants than that on [CMV+Y-sat]-infected plants **(f)**, whereas in a high-density condition, the aphid population size on CMV-infected plants was the lowest among three **(g)**. Source data are provided as a Source Data file.

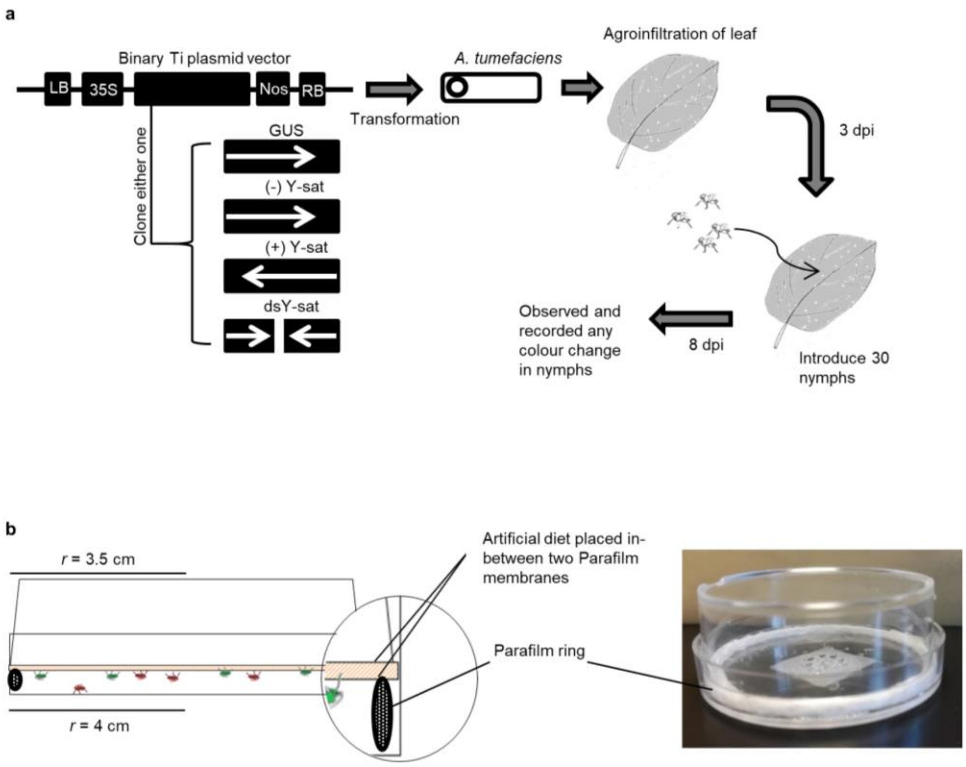

Supplementary Fig. 10 Experimental designs for transient expression of Y-sat and artificial feeding. a, Experimental design for agrobacterium-mediated transient expression of dsRNA (dsY-sat), plus-sense Y-sat (+) and negative-sense Y-sat (-) in *Nicotiana benthamiana* leaves. **b,** Schematic diagram of artificial feeding apparatus to feed Y-sat dsRNA to aphids. See Methods for more detail.

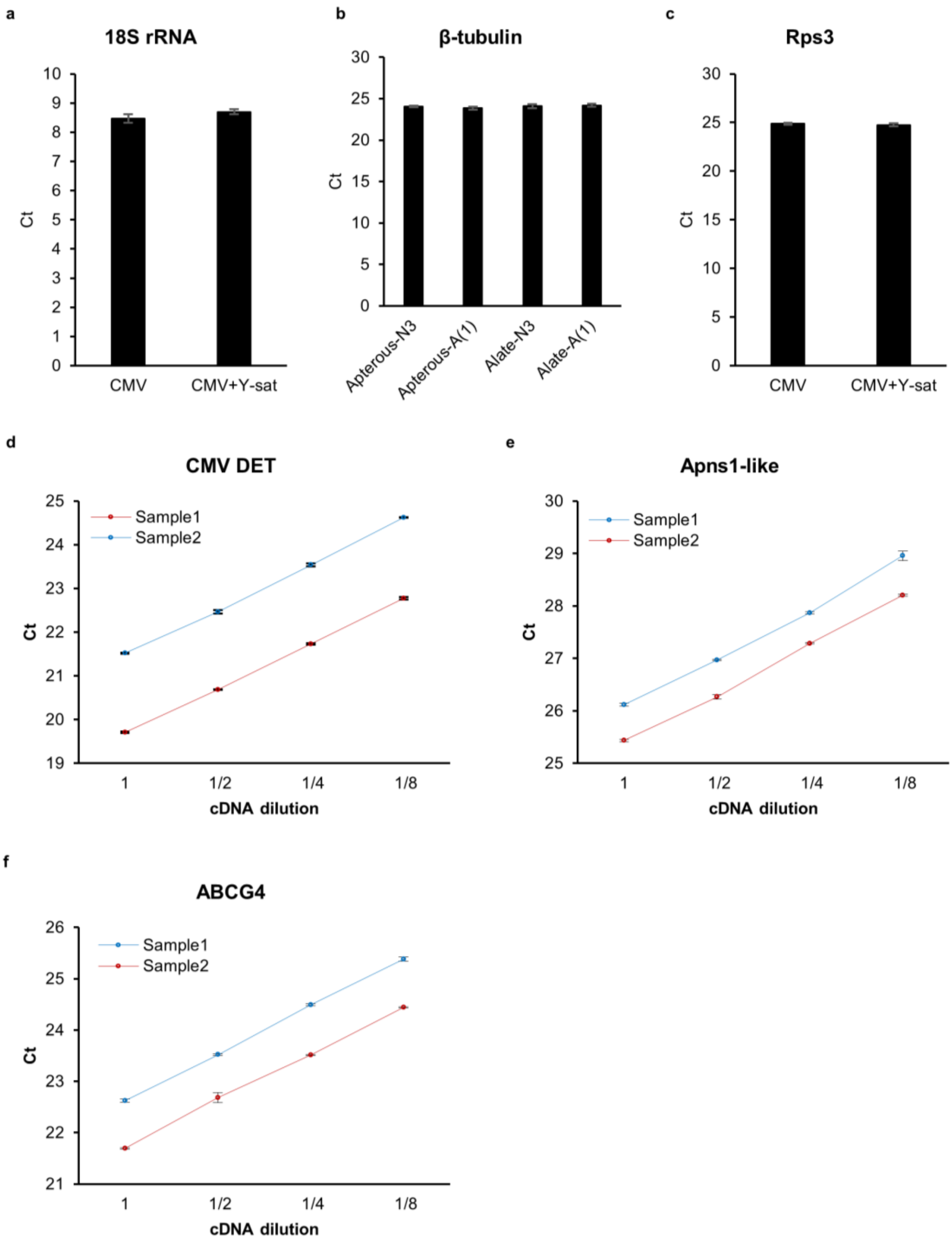

Supplementary Fig. 11 Validation of the primers used for real-time RT-PCR. a-c, Primer validation for the reference genes. Mean (±SE) cycle threshold (Ct) values for **a,** *N. tabacum* 18S rRNA (n = 5), **b,** *M. persicae β-tubulin* (n = 6) and **c,** *M. persicae Rps3* (n = 5) were compared in the conditions which are given in each experiment (e.g., virus infection and aphid developmental stage). **d-f,** Specific and efficient amplification of the target sequence by

primer pair designed for qPCR. Two RNA samples with different concentrations of target mRNA were used. A series of 2-fold dilution of cDNA synthesized from RNA including the target mRNA was first made. The diluted cDNAs were then used for real-time RT-PCR. Mean (±SE) Ct values (*n* = 3) of **d (**CMV RNA3), **e (***Apns1-like*) and **f** (*ABCG4*) are shown. Note that as expected, the amplification of the target sequence was proportional to the concentrations of cDNA.

Supplementary Note 1

Detailed life cycle of *Myzus persicae* focusing on body color and wing formation.

We initially observed a unique body color change from green to red with wing formation in *M. persicae*. To further confirm our observations, we separated and counted the green and red aphids that developed from an individual aphid after 3 weeks and counted their numbers (Methods). We selected 47 red nymphs and 50 green nymphs were separately reared. Among the 50 green nymphs, 46 developed into apterous females, and 4 individuals died at an early stage. Out of 47 red aphids 44 developed into alate females and 3 individuals died at an early stage (Fig. 2b).

In the life cycle of red aphids, the wing buds became visible after the N4 stage; this stage is considered as the early adult [A(1)]. In the subsequent stages, the wing bud grew further, the body color became darker and the body sizes became larger. Therefore the adult stage was further divided into four stages, A(1)-A(4). During the A(3) stage, the wings were further developed but remained unexpanded. At the A(4) stage, aphids with a functioning wing can be seen, and they became blackish and the red body color had faded. During this study, we did not find evidence of sexual reproduction by *M. persicae*. Therefore, only the viviparous life cycle of *M. persicae* is presented in Fig. 2a.

Supplementary Note 2

***M. persicae* colonies were free from *M. persicae* densovirus**

To investigate the involvement of the densovirus genes in Y-sat-mediated wing formation, we first confirmed that the aphid colonies we maintained were free from *M. persicae* densovirus (MpDNV) because MpDNV can influence wing production by *M. persicae*[1] as demonstrated for pea aphids.

Supplementary Note 3

Information of the quantitative real-time RT-PCR used in this study

Total RNA extracts were treated with Recombinant DNase I (Takara), and then used for cDNA synthesis. cDNA was generated from 150 ng of total RNA extract in 10 µl reaction containing oligo dT primer (2.5 µM) and random 6 mers (5 µM) using the PrimeScript RT reagent kit (Takara). Real-time RT-PCR was performed using PowerUp SYBR Green Master Mix (Applied Biosystems) or TB Green Premix Ex Taq II (Tli RNaseH Plus) (Takara) on an ABI StepOne® Real-Time PCR thermocycler (Applied Biosystems, Foster City, USA). For the real-time RT-PCR analysis using PowerUp SYBR Green Master Mix, the PCR amplification was conducted in 10 µl reaction containing 2.5 µl of the diluted cDNAs (5X diluted from the original RT reaction), 5 µl of SYBR green master mix and the gene-specific primer pair (300 nM each). The thermo-cycling condition was as followed; 50°C 2 min, 95°C 2 min and 40 cycles of [95°C 15 sec, 55°C 15 sec and 72°C 30 sec]. For the real-time RT-PCR analysis of using TB Green Premix Ex Taq II (Tli RNaseH Plus), the PCR reaction was conducted in 20 µl reaction containing 2 µl of diluted cDNAs (5X diluted from the original RT reaction), 10 µl of TB Green Premix Ex Taq II (Tli RNaseH Plus), 0.4 µl of ROX Reference Dye (50X) and the gene-specific primer pair (400 nM each). The thermo-cycling condition was as followed; 95°C 30 sec and 40 cycles of [95°C 5 sec, 60°C 34 sec]. All the primers used in real-time RT-PCRs were validated (Supplementary Fig. 11). The data of real-time RT-PCR were analyzed via the 2^(-ΔCt) method[2]: Relative gene expression/viral RNA accumulation = 2^(Ct of reference gene – Ct of viral RNA/target gene).

Supplementary Methods 1 Summary of the results of the statistical analyses for all Figures

Fig. 1b

Unpaired t test	
P value	0.0037
P value summary	**
Significantly different (P < 0.05)?	Yes
One- or two-tailed P value?	Two-tailed
t, df	t=4.054, df=8

Fig. 1c

> chisq.test(matrix(c(22, 13, 8, 17), ncol=2, byrow=T))

Pearson's Chi-squared test with Yates' continuity correction

data: matrix(c(22, 13, 8, 17), ncol = 2, byrow = T)

X-squared = 4.3886, df = 1, p-value = 0.03618

Fig. 1d

> chisq.test(matrix(c(16, 15, 8, 9), ncol=2, byrow=T))

Pearson's Chi-squared test with Yates' continuity correction

data: matrix(c(16, 15, 8, 9), ncol = 2, byrow = T)

X-squared = 0, df = 1, p-value = 1

Fig. 1f top

ANOVA summary	
F	0.5572
P value	0.5914
P value summary	ns
Significant diff. among means (P < 0.05)?	No
R squared	0.1102

Tukey's multiple comparisons test	Mean Diff.	95.00% CI of diff.	Significant?	Summary
Healthy vs. CMV-O	2.750	-4.533 to 10.03	No	ns
Healthy vs. CMV-O+Ysat	1.500	-5.783 to 8.783	No	ns
CMV-O vs. CMV-O+Ysat	-1.250	-8.533 to 6.033	No	ns

Fig. 1f bottom

ANOVA summary	
F	0.07800
P value	0.9256
P value summary	ns

Significant diff. among means (P < 0.05)?	No
R squared	0.01704

Tukey's multiple comparisons test	Mean Diff.	95.00% CI of diff.	Significant?	Summary
Healthy vs. CMV-O	6.200	-37.64 to 50.04	No	ns
Healthy vs. CMV-O+Ysat	3.200	-40.64 to 47.04	No	ns
CMV-O vs. CMV-O+Ysat	-3.000	-46.84 to 40.84	No	ns

Fig. 1h top

ANOVA summary	
F	0.07388
P value	0.9293
P value summary	Ns
Significant diff. among means (P < 0.05)?	No
R squared	0.01615

Tukey's multiple comparisons test	Mean Diff.	95.00% CI of diff.	Significant?	Summary
Healthy vs. CMV-L	1.000	-10.10 to 12.10	No	ns
Healthy vs. CMV-L+Ysat	1.500	-9.595 to 12.60	No	ns
CMV-L vs. CMV-L+Ysat	0.5000	-10.60 to 11.60	No	ns

Fig. 1h (bottom)

ANOVA summary	
F	2.176
P value	0.1695
P value summary	ns
Significant diff. among means (P < 0.05)?	No
R squared	0.3259

Tukey's multiple comparisons test	Mean Diff.	95.00% CI of diff.	Significant?	Summary
Healthy vs. CMV-L	48.00	-22.47 to 118.5	No	ns
Healthy vs. CMV-L+Ysat	42.75	-27.72 to 113.2	No	ns
CMV-L vs. CMV-L+Ysat	-5.250	-75.72 to 65.22	No	ns

Fig. 3a (left, CMV vs [CMV+Y-sat])
Chi-square test
> # 1 hour total
> chisq.test(c(44, 83), p=c(1/2, 1/2))

Chi-squared test for given probabilities
data: c(44, 83)
X-squared = 11.976, df = 1, p-value = 0.0005388

> # 2 hour total
> chisq.test(c(38, 88), p=c(1/2, 1/2))
Chi-squared test for given probabilities
data: c(38, 88)
X-squared = 19.841, df = 1, p-value = 8.415e-06

Hypothesis test for one-sample proportion
> # 1 hour total
> prop.test(x=83,n=127, p=0.5, correct=FALSE, alternative="greater")

1-sample proportions test without continuity correction

data: 83 out of 127, null probability 0.5
X-squared = 11.976, df = 1, p-value = 0.0002694
alternative hypothesis: true p is greater than 0.5
95 percent confidence interval:
0.5815417 1.0000000
sample estimates:
p
0.6535433

> # 2 hour total
> prop.test(x=88,n=126, p=0.5, correct=FALSE, alternative="greater")

1-sample proportions test without continuity correction

data: 88 out of 126, null probability 0.5
X-squared = 19.841, df = 1, p-value = 4.207e-06
alternative hypothesis: true p is greater than 0.5
95 percent confidence interval:
0.6275699 1.0000000
sample estimates:
p
0.6984127

Fig. 3a (right, Healthy vs [CMV+Y-sat])
Chi-Square test
> # 1 hour total
> chisq.test(c(33, 67), p=c(1/2, 1/2))
Chi-squared test for given probabilities

data: c(33, 67)
X-squared = 11.56, df = 1, p-value = 0.0006739

> # 2 hour total
> chisq.test(c(44, 72), p=c(1/2, 1/2))
        Chi-squared test for given probabilities
data: c(44, 72)
X-squared = 6.7586, df = 1, p-value = 0.00933

Hypothesis test for one-sample proportion
> # 1 hour total
> prop.test(x=67,n=100, p=0.5, correct=FALSE, alternative="greater")

1-sample proportions test without continuity correction

data: 67 out of 100, null probability 0.5
X-squared = 11.56, df = 1, p-value = 0.0003369
alternative hypothesis: true p is greater than 0.5
95 percent confidence interval:
 0.5890729 1.0000000
sample estimates:
   p
0.67

> # 2 hour total
>prop.test(x=72,n=116, p=0.5, correct=FALSE, alternative="greater")

        1-sample proportions test without continuity correction

data: 72 out of 116, null probability 0.5
X-squared = 6.7586, df = 1, p-value = 0.004665
alternative hypothesis: true p is greater than 0.5
95 percent confidence interval:
 0.5446341 1.0000000
sample estimates:
        p
0.6206897

Fig. 3b
Chi-Square test
Choice vs No choice
> # 1 hour total
> chisq.test(c(30, 150), p=c(1/2, 1/2))
        Chi-squared test for given probabilities
data: c(30, 150)
X-squared = 80, df = 1, p-value < 2.2e-16

> # 2 hour total
> chisq.test(c(30, 150), p=c(1/2, 1/2))
Chi-squared test for given probabilities
data: c(30, 150)
X-squared = 80, df = 1, p-value < 2.2e-16

CMV vs [CMV+Y-sat]
> # 1 hour total
> chisq.test(c(16, 14), p=c(1/2, 1/2))
Chi-squared test for given probabilities
data: c(16, 14)
X-squared = 0.13333, df = 1, p-value = 0.715

> # 2 hour total
> chisq.test(c(17, 13), p=c(1/2, 1/2))
Chi-squared test for given probabilities
data: c(17, 13)
X-squared = 0.53333, df = 1, p-value = 0.4652

Hypothesis test for one-sample proportion
Choice vs no choice
> # 1 hour total
> prop.test(x=150,n=180, p=0.5, correct=FALSE, alternative="greater")

1-sample proportions test without continuity correction

data: 150 out of 180, null probability 0.5
X-squared = 80, df = 1, p-value < 2.2e-16
alternative hypothesis: true p is greater than 0.5
95 percent confidence interval:
0.7827786 1.0000000
sample estimates:
p
0.8333333

> # 2 hour total
> prop.test(x=150,n=180, p=0.5, correct=FALSE, alternative="greater")

1-sample proportions test without continuity correction

data: 150 out of 180, null probability 0.5
X-squared = 80, df = 1, p-value < 2.2e-16
alternative hypothesis: true p is greater than 0.5
95 percent confidence interval:
0.7827786 1.0000000

sample estimates:
p
0.8333333

[CMV+Ysat] vs CMV
> # 1 hour total
> prop.test(x=16,n=30, p=0.5, correct=FALSE, alternative="greater")

1-sample proportions test without continuity correction

data: 16 out of 30, null probability 0.5
X-squared = 0.13333, df = 1, p-value = 0.3575
alternative hypothesis: true p is greater than 0.5
95 percent confidence interval:
0.3870601 1.0000000
sample estimates:
p
0.5333333

> # 2 hour total
> prop.test(x=17,n=30, p=0.5, correct=FALSE, alternative="greater")

1-sample proportions test without continuity correction

data: 17 out of 30, null probability 0.5
X-squared = 0.53333, df = 1, p-value = 0.2326
alternative hypothesis: true p is greater than 0.5
95 percent confidence interval:
0.4185199 1.0000000
sample estimates:
p
0.5666667

Fig. 3c (left to right)
Chi-Square test
[CMV+Y-sat] vs CMV
> chisq.test(c(10, 11), p=c(1/2, 1/2))
Chi-squared test for given probabilities
data: c(10, 11)
X-squared = 0.047619, df = 1, p-value = 0.8273

[CMV+Y-sat] vs Healthy
> chisq.test(c(8, 6), p=c(1/2, 1/2))
Chi-squared test for given probabilities
data: c(8, 6)

X-squared = 0.28571, df = 1, p-value = 0.593

CMV vs Healthy

```
> chisq.test(c(5, 7), p=c(1/2, 1/2))
        Chi-squared test for given probabilities
data:  c(5, 7)
X-squared = 0.33333, df = 1, p-value = 0.5637
```

Hypothesis test for one-sample proportion

[CMV+Y-sat] vs CMV

```
> prop.test(x=10,n=21, p=0.5, correct=FALSE, alternative="greater")

        1-sample proportions test without continuity correction

data:  10 out of 21, null probability 0.5
X-squared = 0.047619, df = 1, p-value = 0.5864
alternative hypothesis: true p is greater than 0.5
95 percent confidence interval:
 0.310161 1.000000
sample estimates:
        p
0.4761905
```

[CMV+Y-sat] vs Healthy

```
> prop.test(x=8,n=14, p=0.5, correct=FALSE, alternative="greater")

        1-sample proportions test without continuity correction

data:  8 out of 14, null probability 0.5
X-squared = 0.28571, df = 1, p-value = 0.2965
alternative hypothesis: true p is greater than 0.5
95 percent confidence interval:
 0.3603704 1.0000000
sample estimates:
        p
0.5714286
```

CMV vs Healthy

```
> prop.test(x=5,n=12, p=0.5, correct=FALSE, alternative="greater")

        1-sample proportions test without continuity correction

data:  5 out of 12, null probability 0.5
X-squared = 0.33333, df = 1, p-value = 0.7181
alternative hypothesis: true p is greater than 0.5
95 percent confidence interval:
```

0.2199779 1.0000000
sample estimates:
p
0.4166667

Fig. 3d (left to right)
Chi-Square test
[CMV+Y-sat] vs CMV

> chisq.test(c(7, 9), p=c(1/2, 1/2))
Chi-squared test for given probabilities
data: c(7, 9)
X-squared = 0.25, df = 1, p-value = 0.6171

[CMV+Y-sat] vs Healthy

> chisq.test(c(8, 6), p=c(1/2, 1/2))
Chi-squared test for given probabilities
data: c(8, 6)
X-squared = 0.28571, df = 1, p-value = 0.593

CMV vs Healthy

> chisq.test(c(8, 9), p=c(1/2, 1/2))
Chi-squared test for given probabilities
data: c(8, 9)
X-squared = 0.058824, df = 1, p-value = 0.8084

Hypothesis test for one-sample proportion
[CMV+Y-sat] vs CMV

> prop.test(x=7,n=16, p=0.5, correct=FALSE, alternative="greater")

1-sample proportions test without continuity correction

data: 7 out of 16, null probability 0.5
X-squared = 0.25, df = 1, p-value = 0.6915
alternative hypothesis: true p is greater than 0.5
95 percent confidence interval:
0.257658 1.000000
sample estimates:
p
0.4375

[CMV+Y-sat] vs Healthy

> prop.test(x=8,n=14, p=0.5, correct=FALSE, alternative="greater")

1-sample proportions test without continuity correction

```
data:  8 out of 14, null probability 0.5
X-squared = 0.28571, df = 1, p-value = 0.2965
alternative hypothesis: true p is greater than 0.5
95 percent confidence interval:
 0.3603704 1.0000000
sample estimates:
        p
0.5714286
```

CMV vs Healthy

```
> prop.test(x=8,n=17, p=0.5, correct=FALSE, alternative="greater")

        1-sample proportions test without continuity correction

data:  8 out of 17, null probability 0.5
X-squared = 0.058824, df = 1, p-value = 0.5958
alternative hypothesis: true p is greater than 0.5
95 percent confidence interval:
 0.289634 1.000000
sample estimates:
        p
0.4705882
```

Fig. 3e

Chi-Square test (three samples)

```
> t1 <- matrix(c(213, 26, 341, 1103, 724, 1180), ncol=3, byrow=T)
> chisq.test(t1)
        Pearson's Chi-squared test
data:  t1
X-squared = 133.11, df = 2, p-value < 2.2e-16
```

Chi-Square test (two samples)

[CMV+Y-sat] vs CMV

```
> chisq.test(matrix(c(341, 26, 1180, 724), ncol=2, byrow=T))
        Pearson's Chi-squared test with Yates' continuity correction
data:  matrix(c(341, 26, 1180, 724), ncol = 2, byrow = T)
X-squared = 131.78, df = 1, p-value < 2.2e-16
```

[CMV+Y-sat] vs Healthy

```
> chisq.test(matrix(c(341, 213, 1180, 1103), ncol=2, byrow=T))
        Pearson's Chi-squared test with Yates' continuity correction
data:  matrix(c(341, 213, 1180, 1103), ncol = 2, byrow = T)
X-squared = 17.054, df = 1, p-value = 3.632e-05
```

CMV vs Healthy

```
>  chisq.test(matrix(c(26, 213, 724, 1103), ncol=2, byrow=T))
        Pearson's Chi-squared test with Yates' continuity correction
data:  matrix(c(26, 213, 724, 1103), ncol = 2, byrow = T)
X-squared = 74.306, df = 1, p-value < 2.2e-16
```

Hypothesis test for two-sample proportion

[CMV+Y-sat] vs CMV

```
> prop.test(x = c(341,26), n = c(1521,750),
+ alternative = "greater")

        2-sample test for equality of proportions with continuity correction

data:  c(341, 26) out of c(1521, 750)
X-squared = 131.78, df = 1, p-value < 2.2e-16
alternative hypothesis: greater
95 percent confidence interval:
 0.1677935 1.0000000
sample estimates:
    prop 1     prop 2
0.22419461 0.03466667
```

[CMV+Y-sat] vs Healthy

```
> prop.test(x = c(341,213), n = c(1521,1316),
+ alternative = "greater")

        2-sample test for equality of proportions with continuity correction

data:  c(341, 213) out of c(1521, 1316)
X-squared = 17.054, df = 1, p-value = 1.816e-05
alternative hypothesis: greater
95 percent confidence interval:
 0.03737728 1.00000000
sample estimates:
   prop 1    prop 2
0.2241946 0.1618541
```

CMV vs Healthy

```
> prop.test(x = c(213,26), n = c(1316,750),
+ alternative = "greater")

        2-sample test for equality of proportions with continuity correction
```

```
data:  c(213, 26) out of c(1316, 750)
X-squared = 74.306, df = 1, p-value < 2.2e-16
alternative hypothesis: greater
95 percent confidence interval:
 0.1061504 1.0000000
sample estimates:
    prop 1     prop 2 
0.16185410 0.03466667
```

Fig 3f

Chi-Square test

```
> chisq.test(matrix(c(25, 35, 190, 98), ncol=2, byrow=T))
        Pearson's Chi-squared test with Yates' continuity correction
data:  matrix(c(25, 35, 190, 98), ncol = 2, byrow = T)
X-squared = 11.415, df = 1, p-value = 0.0007284
```

Hypothesis test for two-sample proportion

```
> prop.test(x = c(35,25), n = c(133,215),
+ alternative = "greater")

        2-sample test for equality of proportions with continuity correction

data:  c(35, 25) out of c(133, 215)
X-squared = 11.415, df = 1, p-value = 0.0003642
alternative hypothesis: greater
95 percent confidence interval:
 0.06842246 1.00000000
sample estimates:
   prop 1    prop 2 
0.2631579 0.1162791
```

Fig. 4a

ANOVA summary	
F	10.06
P value	0.0031
P value summary	**
Is there significant matching (P < 0.05)?	Yes
R squared	0.4851

Dunnett's multiple comparisons test	Mean Diff.	95.00% CI of diff.	Significant?	Summary
GUS vs. Ysat +	0.05000	-8.407 to 8.507	No	ns
GUS vs. Ysat -	-1.350	-9.963 to 7.263	No	ns

GUS vs. ds-Ysat	-9.525	-12.85 to -6.203	Yes	**

Fig. 4b

ANOVA summary	
F	8.260
P value	0.0030
P value summary	**
Significant diff. among means (P < 0.05)?	Yes
R squared	0.6737

Tukey's multiple comparisons test	Mean Diff.	95.00% CI of diff.	Significant?	Summary
dsY-sat vs. Y-sat +	11.17	3.377 to 18.95	Yes	**
dsY-sat vs. Y-sat -	10.39	2.604 to 18.18	Yes	**
dsY-sat vs. Wild	10.35	2.562 to 18.14	Yes	**
Y-sat + vs. Y-sat -	-0.7725	-8.561 to 7.016	No	ns
Y-sat + vs. Wild	-0.8150	-8.603 to 6.973	No	ns
Y-sat - vs. Wild	-0.04250	-7.831 to 7.746	No	ns

Fig. 4c

ANOVA summary	
F	7.284
P value	0.0085
P value summary	**
Significant diff. among means (P < 0.05)?	Yes
R squared	0.5483

ANOVA table	SS	DF	MS	F (DFn, DFd)	P value
Treatment (between columns)	337.7	2	168.9	F (2, 12) = 7.284	P=0.0085
Residual (within columns)	278.2	12	23.18		
Total	615.9	14			
Data summary					
Number of treatments (columns)	3				
Number of values	15				

Tukey's multiple comparisons test	Mean Diff.	95.00% CI of diff.	Significant?	Summary
Control vs. Ysat	-9.540	-17.53 to -1.548	Yes	*
Control vs. LYSV	0.9800	-7.012 to 8.972	No	ns

Ysat vs. LYSV	10.52	2.528 to 18.51	Yes	*

Fig. 4d

ANOVA summary	
F	19.95
P value	<0.0001
P value summary	****
Significant diff. among means (P < 0.05)?	Yes
R squared	0.8533

Tukey's multiple comparisons test	Mean Diff.	95.00% CI of diff.	Significant?	Summary
Alate-N3 vs. Apterous-N3	-2.500e-007	-0.3574 to 0.3574	No	ns
Alate-N3 vs. Alate-A(1)	0.6596	0.3022 to 1.017	Yes	****
Alate-N3 vs. Apterous-A(1)	0.6772	0.3198 to 1.035	Yes	****
Alate-N3 vs. Alate-N3	-0.08813	-0.4455 to 0.2693	No	ns
Alate-N3 vs. Apterous-N3	-0.2291	-0.5865 to 0.1283	No	ns
Alate-N3 vs. Alate-A(1)	0.1819	-0.1755 to 0.5393	No	ns
Alate-N3 vs. Apterous-A(1)	-0.02077	-0.3782 to 0.3366	No	ns
Apterous-N3 vs. Alate-A(1)	0.6596	0.3022 to 1.017	Yes	****
Apterous-N3 vs. Apterous-A(1)	0.6772	0.3198 to 1.035	Yes	****
Apterous-N3 vs. Alate-N3	-0.08813	-0.4455 to 0.2693	No	ns
Apterous-N3 vs. Apterous-N3	-0.2291	-0.5865 to 0.1283	No	ns
Apterous-N3 vs. Alate-A(1)	0.1819	-0.1755 to 0.5393	No	ns
Apterous-N3 vs. Apterous-A(1)	-0.02077	-0.3782 to 0.3366	No	ns
Alate-A(1) vs. Apterous-A(1)	0.01761	-0.3398 to 0.3750	No	ns
Alate-A(1) vs. Alate-N3	-0.7477	-1.105 to -0.3903	Yes	****
Alate-A(1) vs. Apterous-N3	-0.8887	-1.246 to -0.5313	Yes	****
Alate-A(1) vs. Alate-A(1)	-0.4776	-0.8350 to -0.1203	Yes	**
Alate-A(1) vs. Apterous-A(1)	-0.6803	-1.038 to -0.3229	Yes	****
Apterous-A(1) vs. Alate-N3	-0.7653	-1.123 to -0.4079	Yes	****
Apterous-A(1) vs. Apterous-N3	-0.9063	-1.264 to -0.5489	Yes	****
Apterous-A(1) vs. Alate-A(1)	-0.4952	-0.8526 to -0.1379	Yes	**
Apterous-A(1) vs. Apterous-A(1)	-0.6979	-1.055 to -0.3405	Yes	****
Alate-N3 vs. Apterous-N3	-0.1410	-0.4984 to 0.2164	No	ns
Alate-N3 vs. Alate-A(1)	0.2700	-0.08734 to 0.6274	No	ns
Alate-N3 vs. Apterous-A(1)	0.06736	-0.2900 to 0.4247	No	ns
Apterous-N3 vs. Alate-A(1)	0.4110	0.05363 to 0.7684	Yes	*
Apterous-N3 vs. Apterous-A(1)	0.2083	-0.1491 to 0.5657	No	ns
Alate-A(1) vs. Apterous-A(1)	-0.2027	-0.5601 to 0.1547	No	ns

Fig.4e (left to right)

Unpaired t test			
P value	0.0122	0.0150	0.8923
P value summary	*	*	ns

Significantly different (P < 0.05)?	Yes	Yes	No
One- or two-tailed P value?	Two-tailed	Two-tailed	Two-tailed
t, df	t=3.543, df=6	t=3.371, df=6	t=0.1413, df=6

Fig.4j

Generalized linear model (GLM) in R

```
> d1
  Treatment red green
1        CT   3    37
2        CT   1    35
3        CT   3    33
4        CT   2    30
5       T73   2    37
6       T73   2    33
7       T73   2    42
8       T73   2    40
> d2 <- glm(cbind(red, green) ~ Treatment, data=d1, family=binomial)
> summary(d2)
Call:
glm(formula = cbind(red, green) ~ Treatment, family = binomial,
    data = d1)
Deviance Residuals:
     Min        1Q    Median        3Q       Max
-0.96118  -0.08861   0.01829   0.22163   0.49283
Coefficients:
             Estimate Std. Error z value Pr(>|z|)
(Intercept)   -2.7081     0.3443  -7.866 3.66e-15 ***
TreatmentT73  -0.2364     0.5001  -0.473    0.636
---
Signif. codes:  0 ‘***’ 0.001 ‘**’ 0.01 ‘*’ 0.05 ‘.’ 0.1 ‘ ’ 1
(Dispersion parameter for binomial family taken to be 1)
    Null deviance: 1.5535  on 7  degrees of freedom
Residual deviance: 1.3295  on 6  degrees of freedom
AIC: 25.919
Number of Fisher Scoring iterations: 4
```

Supplementary Fig. 1

Unpaired t test	
P value	0.0006
P value summary	***
Significantly different (P < 0.05)?	Yes
One- or two-tailed P value?	Two-tailed
t, df	t=4.086, df=20

Supplementary Fig. 2b

ANOVA summary

F	10.29
P value	0.0115
P value summary	*
Significant diff. among means (P < 0.05)?	Yes
R squared	0.7743

Tukey's multiple comparisons test	Mean Diff.	95.00% CI of diff.	Significant?	Summary
Healthy vs. CMV	-14.30	-26.54 to -2.061	Yes	*
Healthy vs. CMV+Y-sat	-16.76	-28.99 to -4.518	Yes	*
CMV vs. CMV+Y-sat	-2.457	-14.70 to 9.782	No	ns

Supplementary Fig. 2d

ANOVA summary

F	12.50
P value	0.003
P value summary	**
Significant diff. among means (P < 0.05)?	Yes
R squared	0.7353

Tukey's multiple comparisons test	Mean Diff.	95.00% CI of diff.	Significant?	Summary
Healthy vs. CMV-O	16.50	3.373 to 29.63	Yes	*
Healthy vs. CMV-O+Ysat	22.75	9.623 to 35.88	Yes	**
CMV-O vs. CMV-O+Ysat	6.250	-6.877 to 19.38	No	ns

Supplementary Fig. 2e

ANOVA summary

F	1.090
P value	0.3769
P value summary	ns
Significant diff. among means (P < 0.05)?	No
R squared	0.1950

Tukey's multiple comparisons test	Mean Diff.	95.00% CI of diff.	Significant?	Summary
Healthy vs. CMV-O	9.500	-85.67 to 104.7	No	ns
Healthy vs. CMV-O+Ysat	47.55	-47.62 to 142.7	No	ns

CMV-O vs. CMV-O+Ysat	38.05	-57.12 to 133.2	No	ns

Supplementary Fig. 2g

ANOVA summary	
F	0.1166
P value	0.8239
P value summary	ns
Significant diff. among means (P < 0.05)?	No
R squared	0.02525

Tukey's multiple comparisons test	Mean Diff.	95.00% CI of diff.	Significant?	Summary
Healthy vs. CMV-L	-0.2500	-12.85 to 12.35	No	ns
Healthy vs. CMV-L+Ysat	1.750	-10.85 to 14.35	No	ns
CMV-L vs. CMV-L+Ysat	2.000	-10.60 to 14.60	No	ns

Supplementary Fig. 2h

ANOVA summary	
F	7.780
P value	0.0259
P value summary	*
Significant diff. among means (P < 0.05)?	Yes
R squared	0.6335

Tukey's multiple comparisons test	Mean Diff.	95.00% CI of diff.	Significant?	Summary
Healthy vs. CMV-L	16.00	-18.02 to 50.02	No	ns
Healthy vs. CMV-L+Ysat	47.25	13.23 to 81.27	Yes	*
CMV-L vs. CMV-L+Ysat	31.25	-2.770 to 65.27	No	ns

Supplementary Fig. 8a (left to right)

ANOVA summary	
F	20.14
P value	<0.0001
P value summary	****
Significant diff. among means (P < 0.05)?	Yes
R squared	0.8545

Tukey's multiple comparisons test	Mean Diff.	95.00% CI of diff.	Significant?	Summary
Alate-N3 vs. Apterous-N3	0.7920	0.06721 to 1.517	Yes	*
Alate-N3 vs. Alate-A(1)	1.658	0.9330 to 2.383	Yes	****
Alate-N3 vs. Apterous-A(1)	1.610	0.8849 to 2.334	Yes	****

Alate-N3 vs. Alate-N3	-0.2021	-0.9269 to 0.5227	No	ns
Alate-N3 vs. Apterous-N3	0.5887	-0.1361 to 1.314	No	ns
Alate-N3 vs. Alate-A(1)	0.3587	-0.3661 to 1.083	No	ns
Alate-N3 vs. Apterous-A(1)	1.154	0.4294 to 1.879	Yes	***
Apterous-N3 vs. Alate-A(1)	0.8657	0.1410 to 1.591	Yes	*
Apterous-N3 vs. Apterous-A(1)	0.8176	0.09286 to 1.542	Yes	*
Apterous-N3 vs. Alate-N3	-0.9941	-1.719 to -0.2693	Yes	**
Apterous-N3 vs. Apterous-N3	-0.2033	-0.9281 to 0.5215	No	ns
Apterous-N3 vs. Alate-A(1)	-0.4333	-1.158 to 0.2914	No	ns
Apterous-N3 vs. Apterous-A(1)	0.3622	-0.3626 to 1.087	No	ns
Alate-A(1) vs. Apterous-A(1)	-0.04810	-0.7729 to 0.6767	No	ns
Alate-A(1) vs. Alate-N3	-1.860	-2.585 to -1.135	Yes	****
Alate-A(1) vs. Apterous-N3	-1.069	-1.794 to -0.3442	Yes	**
Alate-A(1) vs. Alate-A(1)	-1.299	-2.024 to -0.5743	Yes	****
Alate-A(1) vs. Apterous-A(1)	-0.5036	-1.228 to 0.2212	No	ns
Apterous-A(1) vs. Alate-N3	-1.812	-2.536 to -1.087	Yes	****
Apterous-A(1) vs. Apterous-N3	-1.021	-1.746 to -0.2961	Yes	**
Apterous-A(1) vs. Alate-A(1)	-1.251	-1.976 to -0.5262	Yes	***
Apterous-A(1) vs. Apterous-A(1)	-0.4555	-1.180 to 0.2693	No	ns
Alate-N3 vs. Apterous-N3	0.7908	0.06601 to 1.516	Yes	*
Alate-N3 vs. Alate-A(1)	0.5607	-0.1641 to 1.286	No	ns
Alate-N3 vs. Apterous-A(1)	1.356	0.6315 to 2.081	Yes	****
Apterous-N3 vs. Alate-A(1)	-0.2301	-0.9548 to 0.4947	No	ns
Apterous-N3 vs. Apterous-A(1)	0.5655	-0.1593 to 1.290	No	ns
Alate-A(1) vs. Apterous-A(1)	0.7955	0.07074 to 1.520	Yes	*

Supplementary Fig. 8b (left to right)

Unpaired t test				
P value	0.0014	0.0737	0.0029	0.0519
P value summary	**	ns	*	Ns
Significantly different (P < 0.05)?	Yes	No	Yes	No
One- or two-tailed P value?	Two-tailed	Two-tailed	Two-tailed	Two-tailed
t, df	t=5.582, df=6	t=2.164, df=6	t=3.038, df=6	t=2.419, df=6

Supplementary Fig. 9a

ANOVA summary	
F	0.02991
P value	0.9707
P value summary	ns

Significant diff. among means (P < 0.05)?	No
R squared	0.009873

Supplementary Fig. 9b (left)

ANOVA summary	
F	0.5373
P value	0.5977
P value summary	ns
Significant diff. among means (P < 0.05)?	No
R squared	0.08219

Supplementary Fig. 9b (right)

ANOVA summary	
F	0.6858
P value	0.5392
P value summary	ns
Significant diff. among means (P < 0.05)?	No
R squared	0.1861

Supplementary Fig. 9c (left)

ANOVA summary	
F	0.1436
P value	0.8677
P value summary	ns
Significant diff. among means (P < 0.05)?	No
R squared	0.02337

Supplementary Fig. 9c (right)

ANOVA summary	
F	0.2648
P value	0.7759
P value summary	ns
Significant diff. among means (P < 0.05)?	No
R squared	0.08111

Supplementary Fig. 9d

P value and statistical significance	
Test	Chi-square
Chi-square, df	1.672, 2
P value	0.4335
P value summary	ns
One- or two-sided	NA

Statistically significant (P < 0.05)?	No

Supplementary Fig. 9f

ANOVA summary	
F	50.05
P value	<0.0001
P value summary	****
Significant diff. among means (P < 0.05)?	Yes
R squared	0.4408

Tukey's multiple comparisons test	Mean Diff.	95.00% CI of diff.	Below threshold?	Summary
Healthy vs. CMV-O	-0.02505	-0.03126 to -0.01883	Yes	****
Healthy vs. CMV-O+Y-sat	-0.008100	-0.01461 to -0.001590	Yes	*
CMV-O vs. CMV-O+Y-sat	0.01695	0.01100 to 0.02289	Yes	****

Supplementary Fig. 9g

ANOVA summary	
F	5.063
P value	0.0336
P value summary	*
Significant diff. among means (P < 0.05)?	Yes
R squared	0.5294

Tukey's multiple comparisons test	Mean Diff.	95.00% CI of diff.	Significant?	Summary
Healthy vs. CMV-O	32.25	-6.497 to 71.00	No	ns
Healthy vs. CMV+Y-sat	-10.00	-48.75 to 28.75	No	ns
CMV-O vs. CMV+Y-sat	-42.25	-81.00 to -3.503	Yes	*

Supplementary Methods 2 Code for small RNA size distribution in language C

```
#define _CRT_SECURE_NO_WQRNINGS
#pragma warning(suppress : 4996)
#pragma warning(disable : 4996)
#include <stdio.h>
#include <string.h>
int main()
{
    FILE* fi;
    char name[100];
    int num;
    int n[19] = { 0 };
    if ((fi = fopen("input.txt", "rt")) == NULL) {
        puts("input.txt- no input file.\n");
        return -1;
    }
    int i = 1;
    while (fscanf(fi, " %s ", name) != EOF)
    {
        if (i % 4 == 2)
        {
           num = strlen(name);
            if (num == 18)
            {
                n[1]++;
            }
            if (num == 19)
            {
                n[2]++;
            }
            if (num == 20)
            {
                n[3]++;
            }
            if (num == 21)
            {
                n[4]++;
            }
            if (num == 22)
            {
                n[5]++;
            }
            if (num == 23)
            {
                n[6]++;
            }
            if (num == 24)
            {
                n[7]++;
```

```
        }
        if (num == 25)
        {
            n[8]++;
        }
        if (num == 26)
        {
            n[9]++;
        }
        if (num == 27)
        {
            n[10]++;
        }
        if (num == 28)
        {
            n[11]++;
        }
        if (num == 29)
        {
            n[12]++;
        }
        if (num == 30)
        {
            n[13]++;
        }
        if (num == 31)
        {
            n[14]++;
        }
        if (num == 32)
        {
            n[15]++;
        }
        if (num == 33)
        {
            n[16]++;
        }
        if (num == 34)
        {
            n[17]++;
        }
        if (num == 35)
        {
            n[18]++;
        }
    }
    i++;
}
for (int p = 1; p < 19; p++)
{
```

```
        printf("%d = %d\n", p + 17, n[p]);
    }
    fclose(fi);
    return 0;
}
```

Supplementary References

1. Pinheiro, P. V. *et al.* Plant viruses transmitted in two different modes produce differing effects on small RNA-mediated processes in their aphid vector. *Phytobiomes J.* **3**, 71–81 (2019).

2. Livak K. J & Schmittgen T. D. Analysis of relative gene expression data using real-time quantitative PCR and the 2(-Delta Delta C(T)) Method. *Methods* **25**, 402–408 (2001).
