# Supplementary material for: A plant virus satellite RNA directly accelerates wing formation in its insect vector for spread
Source: Nat Commun. 2021 Dec 6;12:7087. doi: 10.1038/s41467-021-27330-4 (PMC8648847; doi:10.1038/s41467-021-27330-4)
Supplement: Supplementary file 2 — Reporting Summary [file 41467_2021_27330_MOESM2_ESM.pdf]

nature research

Corresponding author(s): Chikara Masuta
Last updated by author(s): Nov 9, 2021

Reporting Summary

Nature Research wishes to improve the reproducibility of the work that we publish. This form provides structure for consistency and transparency in reporting. For further information on Nature Research policies, see our Editorial Policies and the Editorial Policy Checklist.

Statistics

For all statistical analyses, confirm that the following items are present in the figure legend, table legend, main text, or Methods section.

n/a	Confirmed	
[ ]	[x]	The exact sample size (*n*) for each experimental group/condition, given as a discrete number and unit of measurement
[x]	[ ]	A statement on whether measurements were taken from distinct samples or whether the same sample was measured repeatedly
[ ]	[x]	The statistical test(s) used AND whether they are one- or two-sided<br>*Only common tests should be described solely by name; describe more complex techniques in the Methods section.*
[x]	[ ]	A description of all covariates tested
[x]	[ ]	A description of any assumptions or corrections, such as tests of normality and adjustment for multiple comparisons
[ ]	[x]	A full description of the statistical parameters including central tendency (e.g. means) or other basic estimates (e.g. regression coefficient) AND variation (e.g. standard deviation) or associated estimates of uncertainty (e.g. confidence intervals)
[ ]	[x]	For null hypothesis testing, the test statistic (e.g. *F*, *t*, *r*) with confidence intervals, effect sizes, degrees of freedom and *P* value noted<br>*Give P values as exact values whenever suitable.*
[x]	[ ]	For Bayesian analysis, information on the choice of priors and Markov chain Monte Carlo settings
[x]	[ ]	For hierarchical and complex designs, identification of the appropriate level for tests and full reporting of outcomes
[x]	[ ]	Estimates of effect sizes (e.g. Cohen's *d*, Pearson's *r*), indicating how they were calculated

Our web collection on statistics for biologists contains articles on many of the points above.

Software and code

Policy information about availability of computer code

Data collection	No software was used
Data analysis	GraphPad Prism (ver. 8.4.3), SAMtools (ver 1.2), C in Microsoft Visual Studio 2019 (ver 16.4.2), Image J (ver 1.52a), Sickle (ver. 1.33), R (ver 3.6.3)

For manuscripts utilizing custom algorithms or software that are central to the research but not yet described in published literature, software must be made available to editors and reviewers. We strongly encourage code deposition in a community repository (e.g. GitHub). See the Nature Research guidelines for submitting code & software for further information.

Data

Policy information about availability of data

All manuscripts must include a data availability statement. This statement should provide the following information, where applicable:
- Accession codes, unique identifiers, or web links for publicly available datasets
- A list of figures that have associated raw data
- A description of any restrictions on data availability

All data generated or analysed in this study, which support the findings of this study, are included in this published article and its supplementary information.

Field-specific reporting

Life sciences study design

All studies must disclose on these points even when the disclosure is negative.

Sample size	No sample size calculation was performed to predict the sample size. Sample sizes were determined based on magnitude and consistency of the measurable deference in the statistical model used. For the attraction bio-assay over 100 aphids were used to ensure the data robustness.
Data exclusions	No data were excluded.
Replication	All attempts of replication were successful. Data of replicated experiments were given in the text.
Randomization	Allocation to experimental groups was done randomly.
Blinding	Blinding was not relevant or not possible for most of the studies. The sRNA analysis was done without prior information on tested genes or sequence information. All aphids and plant materials were selected totally randomly for the experiments. Conclusions were based on the comparable results, therefore blinding was not relevant. Persons who handle the samples were aware on the treatments.

Reporting for specific materials, systems and methods

We require information from authors about some types of materials, experimental systems and methods used in many studies. Here, indicate whether each material, system or method listed is relevant to your study. If you are not sure if a list item applies to your research, read the appropriate section before selecting a response.

Materials & experimental systems

n/a	Involved in the study	
☐	☒	Antibodies
☒	☐	Eukaryotic cell lines
☒	☐	Palaeontology and archaeology
☐	☒	Animals and other organisms
☒	☐	Human research participants
☒	☐	Clinical data
☒	☐	Dual use research of concern

Methods

n/a	Involved in the study	
☒	☐	ChIP-seq
☒	☐	Flow cytometry
☒	☐	MRI-based neuroimaging

Antibodies

Antibodies used	Anti-Digoxigenin-AP, Fab fragments, Roche, Cat#: 11093274910, Lot#: 11266027
Validation	No primary antibody was used in this study.

Animals and other organisms

Policy information about studies involving animals; ARRIVE guidelines recommended for reporting animal research

Laboratory animals	Not involved.
Wild animals	Not involved.
Field-collected samples	Laboratory reared Myzus persicae (Sulzer) (Insecta: Hemiptera: Aphididae), originally isolated from N. tabacum were used for this study. The M. persicae colony is maintained in the lab for future studies. No field-collected samples were used.
Ethics oversight	No ethical approval was required.

Note that full information on the approval of the study protocol must also be provided in the manuscript.
